# Cost-Effectiveness of Masked Hypertension Screening and Treatment in US Adults With Suspected Masked Hypertension: A Simulation Study

**DOI:** 10.1093/ajh/hpac071

**Published:** 2022-06-05

**Authors:** Matthew B Green, Daichi Shimbo, Joseph E Schwartz, Adam P Bress, Jordan B King, Paul Muntner, James P Sheppard, Richard J McManus, Ciaran N Kohli-Lynch, Yiyi Zhang, Steven Shea, Andrew E Moran, Brandon K Bellows

**Affiliations:** Department of Medicine, Columbia University Irving Medical Center, New York, New York, USA; Department of Medicine, Columbia University Irving Medical Center, New York, New York, USA; Department of Medicine, Columbia University Irving Medical Center, New York, New York, USA; Department of Psychiatry and Behavioral Health, Stony Brook University, Stony Brook, New York, USA; Department of Population Health Sciences, University of Utah, Salt Lake City, Utah, USA; Department of Population Health Sciences, University of Utah, Salt Lake City, Utah, USA; Department of Epidemiology, University of Alabama at Birmingham, Birmingham, Alabama, USA; Nuffield Department of Primary Care Health Sciences, University of Oxford, Oxford, UK; Nuffield Department of Primary Care Health Sciences, University of Oxford, Oxford, UK; Center for Health Services and Outcomes Research, Institute of Public Health and Medicine, Northwestern Feinberg School of Medicine, Northwestern University, Chicago, Illinois,USA; Department of Medicine, Columbia University Irving Medical Center, New York, New York, USA; Department of Medicine, Columbia University Irving Medical Center, New York, New York, USA; Department of Medicine, Columbia University Irving Medical Center, New York, New York, USA; Department of Medicine, Columbia University Irving Medical Center, New York, New York, USA

**Keywords:** ambulatory blood pressure monitoring, blood pressure, cost-effectiveness, home blood pressure monitoring, hypertension, masked hypertension

## Abstract

**BACKGROUND:**

Recent US blood pressure (BP) guidelines recommend using ambulatory BP monitoring (ABPM) or home BP monitoring (HBPM) to screen adults for masked hypertension. However, limited evidence exists of the expected long-term effects of screening for and treating masked hypertension.

**METHODS:**

We estimated the lifetime health and economic outcomes of screening for and treating masked hypertension using the Cardiovascular Disease (CVD) Policy Model, a validated microsimulation model. We simulated a cohort of 100,000 US adults aged ≥20 years with suspected masked hypertension (i.e., office BP 120–129/<80 mm Hg, not taking antihypertensive medications, without CVD history). We compared usual care only (i.e., no screening), usual care plus ABPM, and usual care plus HBPM. We projected total direct healthcare costs (2021 USD), quality-adjusted life years (QALYs), and incremental cost-effectiveness ratios. Future costs and QALYs were discounted 3% annually. Secondary outcomes included CVD events and serious adverse events.

**RESULTS:**

Relative to usual care, adding masked hypertension screening and treatment with ABPM and HBPM was projected to prevent 14.3 and 20.5 CVD events per 100,000 person-years, increase the proportion experiencing any treatment-related serious adverse events by 2.7 and 5.1 percentage points, and increase mean total costs by $1,076 and $1,046, respectively. Compared with usual care, adding ABPM was estimated to cost $85,164/QALY gained. HBPM resulted in lower QALYs than usual care due to increased treatment-related adverse events and pill-taking disutility.

**CONCLUSIONS:**

The results from our simulation study suggest screening with ABPM and treating masked hypertension is cost-effective in US adults with suspected masked hypertension.

## Graphical Abstract

**Graphical Abstract:**
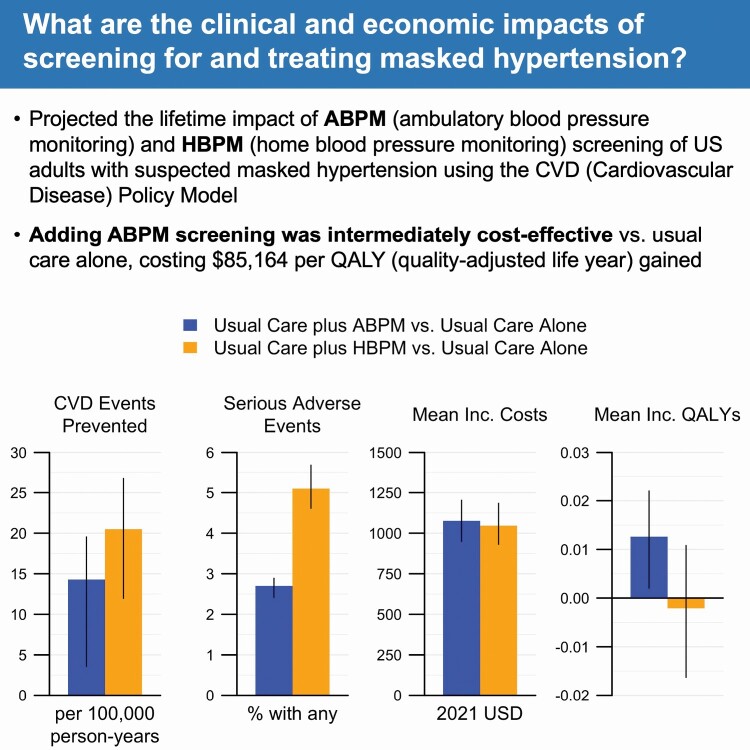

Masked hypertension is defined as having high out-of-office blood pressure (BP) without high office BP among individuals not taking antihypertensive medication.^1^ It is estimated that 17.1 million adults in the United States have masked hypertension.^2^ The risk of incident cardiovascular disease (CVD) and mortality associated with masked hypertension is similar to that of sustained hypertension (both office and out-of-office BP ≥130/80 mm Hg), nearly 2 times that of sustained normotension (both office BP and out-of-office BP <130/80 mm Hg).^2–6^ Using office BP measurements alone will not identify individuals with masked hypertension.

Several BP guidelines recommend screening for masked hypertension among adults without high office BP and treatment for those diagnosed with masked hypertension.^1,7^ The 2017 American College of Cardiology/American Heart Association (ACC/AHA) BP guideline stated it is reasonable to screen for masked hypertension in adults not taking an antihypertensive medication and with office BP readings of 120–129 mm Hg/75–79 mm Hg (Class IIa recommendation).^1^ Treatment with antihypertensive medication is associated with a significant reduction in CVD risk in those with high office BP, but there are currently no randomized clinical trials that have evaluated the effect of treating masked hypertension on CVD outcomes.^8–12^ Despite this, the 2017 ACC/AHA BP guideline stated that initiation of antihypertensive medication for those with masked hypertension is reasonable based on current observational study evidence (Class IIa recommendation).^1^

Out-of-office BP can be measured using ambulatory BP monitoring (ABPM) or home BP monitoring (HBPM).^1,12^ ABPM automatically measures BP, typically every 20–30 minutes, over a 24-hour period, including during the sleep period. HBPM requires an individual to measure their BP in the morning and at night over a minimum of 3 days. ABPM is often considered the reference standard for hypertension diagnosis, but BP guidelines also support the use of HBPM, which is less expensive, and more readily accessible than ABPM.^1,12,13^

Despite recommendations from BP guidelines to screen for masked hypertension using out-of-office BP monitoring, the expected long-term impact of screening for and treating masked hypertension on the risk of incident CVD events, risk of medication-related serious adverse events, and healthcare costs are currently unknown. This study seeks to estimate the lifetime cost-effectiveness of masked hypertension screening and treatment compared with usual care.

## METHODS

### Model overview

We used a microsimulation version of the CVD Policy Model, an established computer simulation of coronary heart disease (CHD) and stroke incidence and prevalence in US adults.^14–17^ In the simulation, individuals started with no history of CVD and each year were at risk of experiencing CHD, stroke, both CHD and stroke, and dying from CVD or non-CVD-related causes (Supplementary Figure S1 online). Individuals were simulated until age 89 years or death.^17^

The simulation model and key inputs used to conduct this research are available upon reasonable request and approval by the model team. Interested researchers can submit a research proposal and collaboration plan to Dr. Bellows and will be required to sign a Creative Commons agreement.

### Population

The model was populated with individuals from National Health and Nutrition Examination Survey (NHANES) 1999–2014 cycles who were matched to participants from the National Heart, Lung and Blood Institute Pooled Cohorts Study (NHLBI-PCS) for whom lifetime CVD risk factor trajectories (i.e., systolic BP [SBP], diastolic BP [DBP], high-density lipoprotein cholesterol, low-density lipoprotein cholesterol, total cholesterol, current smoker, former smoker, cigarettes per day, creatinine, estimated glomerular filtration rate, and diabetes mellitus) have been developed.^14,15,18–21^ Of these, individuals aged 20 years or older with suspected masked hypertension (i.e., mean of 3 SBP/DBP measurements 120–129 mm Hg/<80 mm Hg) with no history of CVD and no self-reported use of antihypertensive medications were included (*n* = 1,223).^1^ To achieve stable cost-effectiveness estimates, nationally representative cohorts of 100,000 US adults were simulated.

### Screening strategies

**Figure 1.**
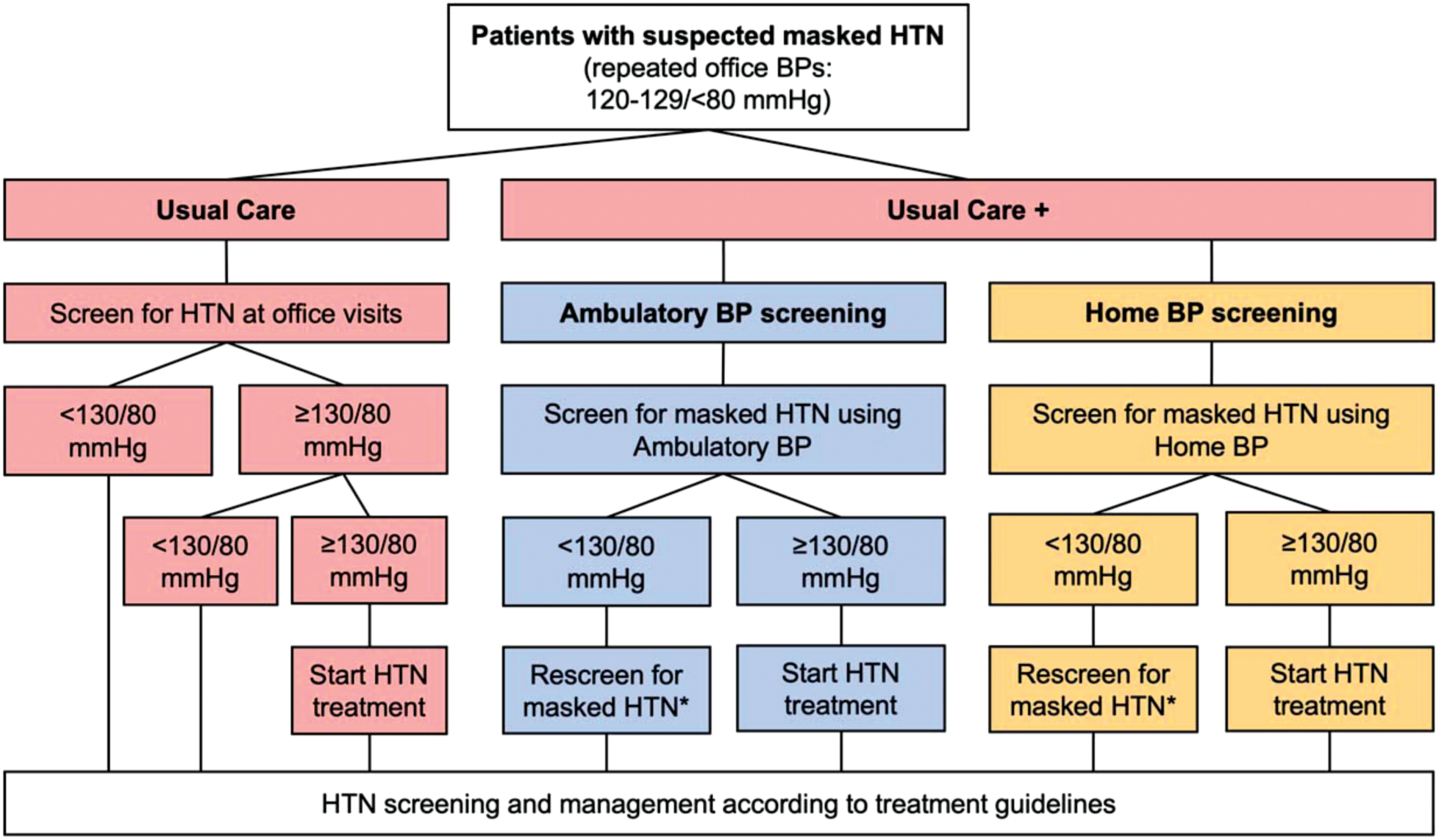
Masked hypertension screening algorithms. Abbreviations: BP, blood pressure; HTN, hypertension. *Rescreening occurs every 3 years when aged <40 years and annually when aged ≥40 years.

We evaluated 3 strategies to diagnose and treat masked hypertension (Figure 1). The first was “usual care,” in which patients received annual screening for and treatment of hypertension using measured office BP per the 2017 ACC/AHA guidelines but did not receive any out-of-office BP screening.^1^ The second and third were “usual care plus ABPM” and “usual care plus HBPM,” in which patients received usual care plus screening for masked hypertension using either ABPM or HBPM, respectively. For masked hypertension screening, patients were rescreened every 3 years when aged <40 years and annually when aged ≥40 years.^22^ Other screening intervals were evaluated in sensitivity analyses.

### Model inputs

#### Blood pressure

The BP components included in the model are described in Table 1. The *untreated* “true” office BP represents an individual’s underlying office BP, without measurement error, had they not been treated with antihypertensive medication at any time in their life. It is derived from the NHLBI-PCS lifetime CVD risk factor trajectories.^14,15,18–21^ The “true” office BP is the treatment-adjusted office BP of an individual that applies the estimated BP reduction based on number of half- and full-standard dose medications used and adherence (Supplementary Table S1 online).^14,23^ To account for uncertainty in office BP measurements, error in BP measurements was incorporated as the difference between the measured office BP and the true office BP.^14,24^ All antihypertensive treatment was assumed to start with a half-standard dose and was subsequently titrated according to measured office BP.

**Table 1.** Blood pressure variables included in the model

Model blood pressure parameter	Definition	Source	Use in simulation
*Untreated* “true” office BP	Underlying office BP if no antihypertensive medications were ever used by patient	Projected trajectory over a lifetime using imputation analysis from NHANES and NHLBI Pooled Cohorts Study^14,19–21^	Used to determine incident CVD event risk before accounting for antihypertensive medication use and masked hypertension status
*Untreated* out-of-office BP	Underlying out-of-office BP if no antihypertensive medications were used	Obtained by applying PROOF-BP algorithm to the untreated “true” BP^25,26^	Used with untreated “true” BP to determine “true” masked hypertension status
“True” office BP	Office BP that accounts for antihypertensive medication use	Obtained by applying the expected BP reduction based on number of half- and full-standard dose medications used and adherence to regimen to the untreated “true” office BP^14,23^	Used to determine reduction in incident CVD event risk with antihypertensive medication treatment
Observed office BP	BP seen in office by provider	Obtained by applying office BP measurement error to “true” office BP^14,24^	Used by provider to make antihypertensive medication treatment decisions
Observed out-of-office hypertension diagnosis	Out-of-office hypertension diagnosis by provider	Obtained by applying the accuracy (i.e., sensitivity and specificity) of ABPM and HBPM to “true” out-of-office BP^27^	Used by provider to make masked hypertension antihypertensive medication initiation decisions

Abbreviations: ABPM, ambulatory blood pressure monitoring; BP, blood pressure; CVD, cardiovascular disease; HBPM, home blood pressure monitoring; NHANES, National Health and Nutrition Examination Survey; NHLBI, National Heart, Lung and Blood Institute; PROOF-BP, PRedicting Out-of-OFfice Blood Pressure.

As NHANES does not include out-of-office BP measurements, the validated Predicting Out-of-OFfice BP (PROOF-BP) algorithm, which uses office BP readings and patient characteristics, was used to estimate *untreated* out-of-office BP.^25,26^ We used sensitivity, specificity, and the out-of-office BP to determine clinical detection of a high out-of-office BP with ABPM and HBPM screening. In the primary analysis, ABPM was assumed to be the reference standard (100% sensitivity and 100% specificity). The diagnostic accuracy of HBPM was obtained from a meta-analysis comparing HBPM measurements with daytime ABPM measurements at a 130/80 mm Hg threshold (sensitivity 91.8% and specificity 41.4%) and accounted for typical use by patients at home.^27^ Similar to prior analyses, failure to obtain a complete ABP reading was set at 5% and required rescreening.^22,28,29^

#### Risk of CVD events and non-CVD mortality

The annual risk of first ever CVD event and non-CVD death was estimated using competing risks Cox proportional hazards functions derived from the NHLBI-PCS and operationalized using logistic regression (Supplementary Table S2 online).^14,15,18,21^ The probability of an incident CVD event each year was calculated using the *untreated* “true” office BP (Supplementary Methods online). In individuals with masked hypertension, an increased risk of CVD was estimated by pooling hazard ratios for masked hypertension vs. normotension from prior observational studies (HR = 1.77; Table 2 and Supplementary Table S3 online).^3–6^ There is limited evidence regarding the benefits of antihypertensive treatment for masked hypertension. Therefore, the relative risk per 10-mm Hg reduction in SBP from meta-analysis was applied to estimate the risk for CHD (RR = 0.83) and stroke (RR = 0.73) when using antihypertensive treatment.^9^ The probabilities for nonincident CVD events were stratified by age and sex (Supplementary Table S4 online).^15^

**Table 2.** Masked hypertension-related model inputs

Parameter	Mean	Sensitivity analysis	Source
Diagnostic accuracy of out-of-office BP screening			
Sensitivity			Ref. ^27^, ABPM lower bound assumed mean HBPM
ABPM	100.0%	91.8%–100.0%	
HBPM	91.8%	84.4%–95.8%	
Specificity			
ABPM	100.0%	41.4%–100.0%	
HBPM	41.4%	30.1%–53.5%	
Out-of-office BP rescreening interval			
Age <40 years	Every 3 years	1–5	Ref. ^22^
Age ≥40 years	Every year	1–5	Ref. ^22^
CVD risk with masked hypertension			
Hazard ratio	1.77	1.23–2.42	Refs. ^3–6^
Costs			
Usual care physician visit (per visit)^a^	$78	$69–97	CMS Physician fee schedule^b^
Screening visit (cost per year)			CMS Physician fee schedule^b^
ABPM^c^	$48	$42–60	
HBPM^d^	$28	$23–34	
Device			
ABPM	$1,916	$1,495–2,195	Mean of top devices from CardiacDirect^e^
HBPM	$54	$45–63	Mean of top devices from Amazon^f^
Number of patients using device			
ABPM	125.0	62.5–187.5	Ref. ^28^
HBPM	1	52 (purchased by clinics)	Clinical judgment
ABPM failure rate	5.0%	0.0%–10.0%	Ref. ^22^
Device replacement	Every 5 years	2–10	Ref. ^28^, clinical judgment
Quality-of-life			
Pill-taking disutility	0.002	0.000–0.006	Refs. ^14,15^

Abbreviations: ABPM, ambulatory blood pressure monitoring; BP, blood pressure; CMS, Center for Medicare and Medicaid Services; CVD, cardiovascular disease; HBPM, home blood pressure monitoring.

^a^CPT 99213.

^b^Physician fee schedule: www.cms.gov/medicare/physician-fee-schedule/search.

^c^Includes recording, scanning analysis, interpretation, and report (CPT 93784).

^d^Includes initial setup, education (CPT code 99473), and interpretation of results (CPT 99474).

^e^24-Hour ABP Monitors: http://www.cardiacdirect.com/product-category/24-hour-abp-monitors.

^f^Best Sellers in Blood Pressure Monitors: www.amazon.com/Best-Sellers-Health-Personal-Care-Blood-Pressure-Monitors.

#### Risk of medication-related adverse events

Medication-related adverse events were classified as tolerable, intolerable (i.e., requiring discontinuation of antihypertensive medications), or serious (i.e., intolerable and requiring hospitalization).^14^ Serious adverse events had a risk of being fatal.^14^ As in prior analyses, the probabilities of each type of adverse event were associated with the number of full- and half-standard doses of antihypertensive medications used (Supplementary Methods online).^14,30–33^

#### Costs

The cost of masked hypertension screening included the cost of setup, education, device, and interpretation of results. The cost of an ABPM device was the average cost of ABPM devices listed on the CardiacDirect website at the time of the analysis (Supplementary Table S5 online). Each ABPM device was assumed to be purchased by physician offices and could be used by an average of 125 patients per year.^28^ The cost of an HBPM device was estimated by averaging the costs of the top 3 rated HBPM devices on Amazon at the time of the analysis. In the primary analysis, patients were assumed to purchase their own HBPM devices (i.e., the cost of 1 device per patient was included), but this was explored in a scenario analysis. The Centers for Medicare and Medicaid Services Physician fee schedule was used to define costs of physician visits associated with ABPM (CPT code 93784) and HBPM (CPT codes 99473 and 99474). Based on prior analyses and warranty information from manufacturers, ABPM and HBPM devices were assumed to be replaced every 5 years in the primary analysis but this was varied in sensitivity analysis.^28,34^ Antihypertensive medication, acute and chronic CVD event, medication-related adverse event, and background healthcare costs were derived from prior cost-effectiveness analyses (Supplementary Table S6 online).^14,15^

#### Quality of life

We quantified health-related quality of life using quality-adjusted life years (QALYs), which range from one, indicating a year of full health, to zero, indicating death.^35^ The QALYs associated with chronic health states were age stratified and adjusted according to CVD history (Supplementary Table S6 online).^15^ Short-term QALY decrements after CVD events and serious adverse event hospitalizations were also included. In the primary analysis, the decrement to quality of life associated with medication pill-taking was 0.002.^14,15^ These assumptions were explored in scenario analyses.

### Calibration and validation

As in prior analyses, the model was calibrated to match contemporary incidence and total event rates of CHD and stroke, and CVD and all-cause mortality from the Centers for Disease Control and Prevention, National Hospital Discharge Survey, National Inpatient Sample, and National Vital Statistics System, and cross-validated against the original, dynamic population version of the CVD Policy Model (Supplementary Methods and Figure S2 online).^14–17^

### Statistical analysis

Our analysis adhered to recommendations from the Second Panel on Cost-effectiveness in Health and Medicine (Supplementary Tables S7 and S8 online).^36^ The simulation model was developed and performed using TreeAge Pro (version 20.2.1, Williamstown, MA) and other analyses were performed in R (version 4.0.2, Vienna, Austria). The primary outcome was the incremental cost-effectiveness ratio (ICER), defined as the additional cost per QALY gained. Costs (2021 USD) were from a US healthcare sector perspective, which includes all direct medical costs regardless of the payer.^36^ All future costs and QALYs were discounted 3% annually.^36^ We used the cost-effectiveness thresholds recommended by the ACC/AHA, considering an ICER <$50,000/QALY gained highly cost-effective and an ICER ≥$50,000/QALY to <$150,000/QALY gained intermediately cost-effective.^37^ For the primary analysis, 200 probabilistic iterations in which model inputs were randomly sampled from prespecified distributions were used to obtain the mean and 95% uncertainty interval (UI) for costs, QALYs, and the probability of being cost-effective. The rates of CVD events, CVD mortality, and serious adverse events were also projected.

### Sensitivity and scenario analyses

One-way sensitivity analyses were performed by systematically varying model inputs related to masked hypertension across plausible ranges while holding all other parameters constant. The lower and upper bounds for the 1-way sensitivity analyses were derived from 95% confidence intervals or ranges reported in the literature. As the potential benefits and harms of treating masked hypertension are unknown, a 2-way sensitivity analysis assessed the joint impact of the probability of serious adverse events and the relative risk of CVD events per 10-mm Hg reduction in office BP with antihypertensive treatment on cost-effectiveness outcomes. Several scenario analyses were conducted to compare our approach to calculating the expected reduction in CVD event risk and quality of life to those used in prior cost-effectiveness studies (Supplementary Methods online).^22,28,29^ Finally, scenario analyses were performed in which (i) HBPM devices were purchased by physician offices and used by an average of 52 patients per year (i.e., the device cost was divided by 52 to calculate the cost per patient), (ii) HBPM had 100% sensitivity and specificity to detect masked hypertension, and (iii) ABPM had the same accuracy as HBPM (i.e., 91.8% sensitivity and 41.4% specificity).^14,15^

## RESULTS

### Participant characteristics

The simulated cohort was 43.3% female; 47.8% were non-Hispanic White and 19.5% non-Hispanic Black. The mean (SD) baseline age was 42.2 (14.8) years, body mass index was 25.4 (4.3) kg/m^2^, office SBP was 123.9 (2.6) mm Hg, and office DBP was 72.1 (5.5) mm Hg.

### Clinical outcomes

Due to decreased specificity, screening for masked hypertension with HBPM was projected to result in more antihypertensive medications being started within the first year (68.3%) than usual care alone (0.0%) or usual care plus ABPM (28.5%). Screening for masked hypertension was projected to reduce the cumulative incidence of CVD events (Supplementary Figure S3 online). Compared with usual care alone, usual care plus ABPM screening decreased the CVD event rate by 14.3 (95% UI: 3.5–19.6) events per 100,000 person-years and usual care plus HBPM by 20.5 (95% UI: 11.9–26.8) events per 100,000 person-years (Table 3). The proportion of individuals who experienced a serious adverse event increased 2.7 percentage points with usual care plus ABPM (95% UI: 2.4–2.9) and 5.1 percentage points with usual care plus HBPM (95% UI: 4.6–5.7) relative to usual care.

**Table 3.** Lifetime CVD, survival, and adverse event projections

Outcome (95% UI)	Usual care	Usual care plus ABPM	Usual care plus HBPM
CVD (% with ≥1 event)	25.6% (24.0%–26.9%)	25.4% (23.7%–26.8%)	25.1% (23.4%–26.6%)
CVD (total events per 100,000 person-years)	1,057.7 (994.5–1,110.6)	1,043.4 (979.0–1,101.5)	1,037.2 (968.2–1,095.7)
Stroke (total events per 100,000 person-years)	162.3 (154.1–169.3)	156.6 (149.4–162.9)	156.6 (149.4–162.5)
CHD (total events per 100,000 person-years)	895.4 (837.5–949.4)	886.8 (826.5–940)	880.5 (817.8–935.3)
CVD death (total events per 100,000 person-years)	336.3 (316.0–353.4)	330.7 (309.3, 349.1)	330.4 (308.5–348.9)
Survival (mean years)	40.3 (40.2–40.4)	40.3 (40.3–40.4)	40.3 (40.3–40.4)
Serious adverse events (% with ≥1 event)	20.5% (19.8%–21.0%)	23.2% (22.3%–23.9%)	25.6% (24.4%–26.7%)
Serious adverse events (per 100,000 person-years)	589.5 (569.4–607.4)	671.0 (643.8–697.2)	750.8 (712.7–786.7)

Abbreviations: ABPM, ambulatory blood pressure monitoring; CHD, coronary heart disease; CVD, cardiovascular disease; HBPM, home blood pressure monitoring; UI, uncertainty interval.

### Cost-effectiveness

**Figure 2.**
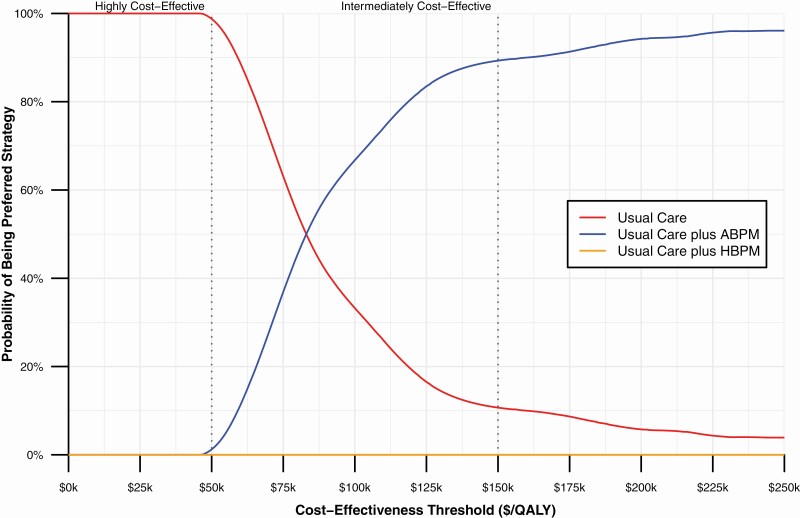
Cost-effectiveness acceptability curve. Abbreviations: ABPM, ambulatory blood pressure monitoring; HBPM, home blood pressure monitoring; QALY, quality-adjusted life year.

Compared with usual care alone, usual care plus ABPM screening increased costs by an average $1,076 (95% UI: $945–1,206) per person over a lifetime and usual care plus HBPM by an average of $1,046 (95% UI: $928–1,187) per person (Table 4 and Supplementary Figure S4 online). Cost differences were driven by increased antihypertensive medication and antihypertensive related adverse events, which were partially offset by savings in CVD events prevented (Supplementary Table S9 online). Usual care plus ABPM gained 0.0126 (95% UI: 0.0019–0.0221) QALYs per person compared with usual care alone. Due to increased serious adverse events and pill-taking disutility, usual care plus HBPM lost 0.0021 (95% UI: −0.0163, 0.0109) QALYs compared with usual care alone. The resulting ICER for usual care plus ABPM vs. usual care alone was $85,164/QALY gained. Usual care plus HBPM was dominated by usual care alone (i.e., usual care plus HBPM was more costly and less effective). At a $150,000/QALY cost-effectiveness threshold, usual care plus ABPM had an 89.5% probability of being cost-effective (Table 4 and Figure 2).

**Table 4.** Lifetime cost, effectiveness, and cost-effectiveness outcomes

Outcomes	Usual care	Usual care plus ABPM	Usual care plus HBPM
Costs (2021 USD)	$199,899	$200,975	$200,945
Incremental costs (95% UI)^a^	—	$1,076 ($945–1,206)	$1,046 ($928–1,187)
QALYs	18.6362	18.6499	18.6341
Incremental QALYs (95% UI)^a^	—	0.0126 (0.0019–0.0221)	−0.0021 (−0.0164, 0.0109)
ICER (Inc. $/QALY gained)^b^	—	$85,164	Dominated
Probability preferred strategy at			
$50,000/QALY	100.0%	0.0%	0.0%
$100,000/QALY	34.0%	66.0%	0.0%
$150,000/QALY	10.5%	89.5%	0.0%

Abbreviations: ABPM, ambulatory blood pressure monitoring; HBPM, home blood pressure monitoring; ICER, incremental cost-effectiveness ratio; QALY, quality-adjusted life year; UI, uncertainty interval.

^a^Relative to usual care.

^b^Relative to next least costly nondominated strategy.

### Sensitivity and scenario analyses

**Figure 3.**
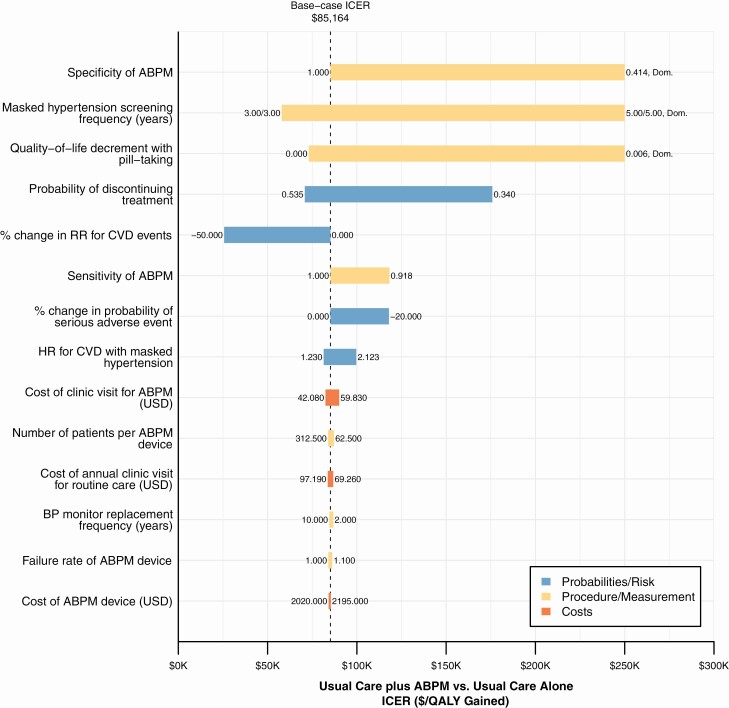
One-way sensitivity analyses of masked hypertension screening and treatment parameters, usual care plus ABPM vs. usual care alone. Notes: The figure shows the change in the ICER for usual care plus ABPM vs. usual care alone (*x*-axis) when independently varying the parameters shown (*y*-axis) across plausible ranges. The dashed line indicates the base-case ICER. At the ends of each bar, the parameter values that resulted in the maximum and minimum ICER are shown. The bars indicates if the parameter was associated with event probabilities/risk, ABPM measurement or procedures, or costs. Parameter values of screening frequency for masked hypertension are shown as aged <40 years/aged ≥40 years. Usual care alone dominated usual care plus ABPM (i.e., usual care cost less and was more effective) when the specificity of ABPM was 41.4% (same as HBPM), the screening frequency for masked hypertension was 5.00/5.00 years, and when pill-taking disutility was set to 0.006; the bars in the plot are cut off at $250,000/QALY gained for presentation. Usual care plus HBPM dominated usual care plus ABPM when pill-taking disutility was ≤0.001. Abbreviations: ABPM, ambulatory blood pressure monitoring; HBPM, home blood pressure monitoring; ICER, incremental cost-effectiveness ratio; QALY, quality-adjusted life year.

The cost-effectiveness of masked hypertension screening was most sensitive to changes in the specificity of ABPM, frequency of masked hypertension screening, and pill-taking disutility (Figure 3). The impact of simultaneous changes to the RR of CVD events and probability of treatment-related serious adverse events are shown in Supplementary Figure S5 online. Usual care plus ABPM was estimated to be highly cost-effective if the RR of CVD events was improved by 20%.

In the scenario analyses that used approaches from prior studies to calculate the expected reduction in CVD event risk and quality of life, a greater number of CVD events were prevented with masked hypertension screening using ABPM (31.1 per 100,000 person-years) and HBPM (43.9 per 100,000 person-years) than in the primary analysis (Supplementary Figure S6 online). Subsequently, usual care plus ABPM became highly cost-effective (ICER $30,269/QALY gained), but the results varied with pill-taking disutility (Supplementary Table S10 online). Assuming HBPM devices were purchased by physician offices and reused by patients had no impact on the cost-effectiveness of usual care plus HBPM (i.e., usual care plus HBPM remained dominated by usual care alone) (Supplementary Table S11 online). When HBPM was assumed to have 100% sensitivity and specificity, usual care plus HBPM dominated usual care plus ABPM and was cost-effective (ICER vs. usual care alone: $79,353/QALY gained). Usual care plus ABPM became dominated by usual care when the sensitivity and specificity were assumed to be the same as HBPM.

## DISCUSSION

In this simulation study, we evaluated the cost-effectiveness of screening for and treating masked hypertension using either ABPM or HBPM in US adults with suspected masked hypertension. Both usual care plus ABPM and usual care plus HBPM screening strategies were projected to decrease CVD events and CVD-related mortality compared with usual care at lifetime incremental costs of screening near $1,000. Adding screening with ABPM to usual care was estimated to cost an average of $85,164/QALY gained over an individual’s lifetime compared with usual care. However, usual care plus HBPM resulted in lower QALYs than usual care due to increased treatment-related adverse events and pill-taking disutility and was not cost-effective. The cost-effectiveness of masked hypertension screening was most sensitive to the specificity of the screening strategy and the screening frequency.

The economic impact of out-of-office BP monitoring has been explored in previous studies, which have shown screening with ABPM to cost less and be more effective than not screening.^22,28,29^ However, these prior studies examined different populations and uses of ABPM compared with our study. Beyhaghi and Viera^22^ examined the use of ABPM for adults aged 21 years and older in a US representative population, including those with high office BP.^1^ Lovibond *et al.*^28^ focused on use of ABPM in those with an office BP ≥140/90 mm Hg, and Monahan *et al.*^29^ on those with an office BP ≥130/80 mm Hg, aged 40 years and older. In contrast, we examined US adults aged 20 years and older without high office BP and with suspected masked hypertension (office BP 120–129/<80 mm Hg, no antihypertensive medication use, and no CVD history), many of whom may have a much lower risk of CVD than the populations studied in prior studies. Additionally, the prior studies used a different approach to estimating QALY reductions with CVD that generally results in a greater quality of life benefit from CVD event prevention than our approach and used a greater CVD event-specific relative risk for individuals on antihypertensive medications than in our analysis.^22,28,29^ When we adopted a similar approach, usual care plus ABPM became highly cost-effective (ICER $30,269/QALY gained).^28^

### Limitations

The results of our study need to be considered in the context of the following potential limitations. While the CVD Policy Model was calibrated to reproduce contemporary CVD event rates and mortality, it is a simulation model with a fixed set of assumptions about the underlying data and real-world clinical practice. Our primary analysis assumed the treatment benefits of antihypertensive medications were based on changes to office SBP and that individuals with masked hypertension receive the same treatment benefit per 10-mm Hg office SBP reduction as individuals with sustained hypertension. This assumption was based on the results from prior studies, but the treatment effect of antihypertensives on out-of-office BP and CVD risk in masked hypertension has not been assessed in any published randomized control trials.^22,28,29^ These assumptions resulted in a modest CVD benefit in our model because office BP is lower in those with masked hypertension than those with sustained hypertension, and meta-analyses show higher starting BPs are associated with greater BP reductions with treatment.^30,31,38^ Additionally, our analysis did not include the reduction in risk of heart failure associated with antihypertensive treatment.^9^ Heart failure is associated with substantial morbidity, mortality, and healthcare costs; therefore, the benefits of antihypertensive treatment are likely underestimated in our analysis.^39,40^ Another potential limitation of our study is the assumption that ABPM is the reference standard (100% sensitivity and specificity) for diagnosing masked hypertension. Recent literature suggests that, compared with 24-hour ABPM, mean BP measured by 1 week of HBPM is more reliable and strongly associated with left ventricular mass index, a reliable measure of end-organ damage.^41^ When HBPM was assumed to have 100% sensitivity and specificity, usual care plus HBPM was projected to be the most cost-effective option (ICER: $79,353/QALY gained). Given this, and that the 2017 ACC/AHA BP guidelines state that HBPM or ABPM may be used to screen for masked hypertension, additional research is needed to better understand the ability of out-of-office screening to detect masked hypertension. Additionally, our analysis did not examine the impact of screening for masked hypertension within specific racial or ethnic groups. Future work is needed to ensure that screening for masked hypertension is equitable and reaches those at highest risk. Finally, while the PROOF-BP algorithm has been shown to accurately predict out-of-office BP, it may lead to some misclassification of participants.

In this simulation analysis, the addition of ABPM to usual care is estimated to reduce CVD morbidity and mortality and be an intermediately cost-effective way to screen for and treat masked hypertension in US adults suspected to have masked hypertension.

## Supplementary Material

hpac071_suppl_Supplementary_Material
