# Supplementary material for: Cost-Effectiveness of Masked Hypertension Screening and Treatment in US Adults With Suspected Masked Hypertension: A Simulation Study
Source: Am J Hypertens. 2022 Jun 5;35(8):752–62. doi: 10.1093/ajh/hpac071 (PMC9340638; doi:10.1093/ajh/hpac071)
Supplement: hpac071_suppl_Supplementary_Material [file hpac071_suppl_supplementary_material.doc]

**SUPPLEMENTAL MATERIAL**

**Title:** Cost-effectiveness of masked hypertension screening and treatment in US adults with suspected masked hypertension.

**Authors:** Matthew B. Green, MPH^1^; Daichi Shimbo, MD^1^; Joseph E. Schwartz, PhD^1,2^; Adam P. Bress, PharmD, MS^3^; Jordan B. King, PharmD, MS^3^; Paul Muntner, PhD^4^; James P. Sheppard, PhD^5^; Richard J. McManus, MA, PhD, MBBS^5^; Ciaran N. Kohli-Lynch, MS^6^; Yiyi Zhang, PhD^1^; Steven Shea, MD, MS^1^; Andrew E. Moran, MD, MPH^1^; Brandon K. Bellows, PharmD, MS^1^

**Affiliations:** (1) Department of Medicine, Columbia University Irving Medical Center, New York, NY, USA; (2) Department of Psychiatry and Behavioral Health, Stony Brook University, Stony Brook, NY, USA; (3) Department of Population Health Sciences, University of Utah, Salt Lake City, UT, USA; (4) Department of Epidemiology, University of Alabama at Birmingham, Birmingham, AL, USA; (5) Nuffield Department of Primary Care Health Sciences, University of Oxford, Oxford, UK; (6) Center for Health Services & Outcomes Research, Northwestern University, Chicago, IL, USA.

**Corresponding Author:**

Brandon K. Bellows, PharmD, MS

Department of Medicine, Columbia University

622 West 168th Street, PH9-105

New York, NY 10032

Tel. 212-305-2569

[bkb2132@cumc.columbia.edu](mailto:bkb2132@cumc.columbia.edu)

**SUPPLEMENTAL TABLE OF CONTENTS**

**Supplemental Methods – page 4-6**

- Approach to Risk of Medication-related Adverse Events
- Alternate Approach to Cardiovascular Disease Risk Reduction with Antihypertensive Treatment
- Alternate Approach to Quality-of-Life Decrements

**Supplemental Tables – pages 7-26**

- Table S1. Hypertension Treatment-Related CVD Policy Model Inputs.
- Table S2. Logistic Risk Functions to Estimate Probability of Incident CVD Events and Non-CVD Mortality in the CVD Policy Model.
- Table S3. CVD Risk Estimates for Masked Hypertension.
- Table S4. Probabilities for Non-Incident CVD Events in the CVD Policy Model.
- Table S5. ABPM and HBPM Device Costs.
- Table S6. CVD Policy Model Cost and Utility Inputs.
- Table S7. Checklist for Cost-Effectiveness Analyses from the Second Panel on Cost-Effectiveness in Health and Medicine.
- Table S8. Formal Health Care Sector Impact Inventory Assessment.
- Table S9. Disaggregated Incremental Costs vs. Usual Care.
- Table S10. Costs, Effectiveness, and Cost-effectiveness Outcomes of Modified Approach to Utility and CVD Risk Reduction with Treatment.
- Table S11. Costs, Effectiveness, and Cost-effectiveness Outcomes of Scenario Analyses.

**Supplemental Figures – pages 27-33**

- Figure S1. Diagram of CVD Policy Model.
- Figure S2. Model Validation and Calibration Results.
- Figure S3. Cumulative Incidence of CVD Events.
- Figure S4. Cost-Effectiveness Scatter Plot (vs. Usual Care).
- Figure S5. Two-Way Sensitivity Analysis of Reducing the Risk of Serious Adverse Events and Relative Risk for CVD Events with Antihypertensive Treatment.
- Figure S6. Cumulative Incidence of CVD Events Using Modified Approach to CVD Risk Reduction with Antihypertensive Treatment.

**References – pages 34-38**

**SUPPLEMENTAL METHODS**

**Probability of Incident Cardiovascular Disease Events**


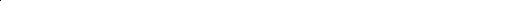


∆BP – change in office blood pressure with antihypertensive treatment, CVD – cardiovascular disease, HR – hazard ratio, HTN – hypertension, P – probability, RR – relative risk, | – “given” (conditional probability)

**Approach to Risk of Medication-related Adverse Events^14^**

As in prior analyses, the probabilities of experiencing any and intolerable adverse events were derived from published meta-analyses.^30,32^ These were weighted by national utilization data from the National Health and Nutrition Examination Survey 1999-2014 cycles to estimate the probability by number of antihypertensive medications used. Pooled data from a published meta-analysis and the Systolic Blood Pressure Intervention Trial (SPRINT) were used to estimate the risk of serious adverse events when using ≤2 vs. >2 full-standard dose antihypertensive medications.^31,33^ The risk of a serious adverse events being fatal was derived from the Agency for Healthcare Research and Quality’s Healthcare Cost and Utilization Project (HCUPnet) tool.

**Calibration and Validation**

The simulation model was calibrated reproduce contemporary rates of coronary heart disease (CHD) and stroke, and cardiovascular disease (CVD) and all-cause mortality. Calibration was performed by setting the alpha parameter of the logisitic regression functions used to predict the risk of incident CHD, incident stroke, and non-CVD death. Multipliers were used for the risk of recurrent events to match the total CHD and stroke event rate targets. The internal validity of the calibration was assessed by visual comparison and regression of the simulated model outputs against the calibration targets. From the regression, an intercept of 0 and slope of 1 would indicate perfect reproduction of the observed targets. Overall, the model was able to reproduce the calibration targets from visual inspection (**Figure S2**). From the regressions, the intercepts ranged from -0.69 to 0.28 and the slopes ranged from 0.89 to 1.12.

**Alternate Approach to Cardiovascular Disease Risk Reduction with Antihypertensive Treatment**

In scenario analyses, the relative risk (RR) of a cardiovascular disease (CVD) event with antihypertensive treatment was modified to be comparable to the approach used in other studies examining the cost-effectiveness of ambulatory blood pressure monitoring (ABPM) and home blood pressure monitoring (HBPM).^22,28,29^

In the base-case analysis, the RR for CVD events was derived from a large meta-analysis and was applied per 10-mmHg reduction in office systolic blood pressure (SBP); 0.82 for coronary heart disease (CHD) events and 0.70 for stroke.^9^ The expected reduction in office SBP was calculated using a published meta-analysis, was dependent upon the untreated office BP, and accounted for patient adherence.^14,23,30,32^ Using this approach, the higher the untreated office BP, the greater the expected reduction in office BP with antihypertensive treatment. For example, a patient with an office SBP of 127 mmHg, would have an approximate 6-mmHg reduction in office SBP when starting one half-standard dose of antihypertensive medication, resulting in an RR of about 0.89 for CHD (i.e., 0.82^(6 mmHg/10 mmHg) = 0.89) and 0.81 for stroke (i.e., 0.70^(6 mmHg/10 mmHg) = 0.81).

In other published studies of the cost-effectiveness of using HBPM and ABPM, the RR was also calculated using meta-analyses, but was either age- and sex-dependent, calculated using the mean office SBP within groups, or was a single value.^22,28,29,32^ Therefore, the RRs for CVD events did not correspond to the amount of office SBP reduction expected with antihypertensive treatment. In a scenario analysis, the same RRs with antihypertensive treatment as Beyhaghi et al. (0.68 for CHD of and 0.63 for stroke) were used, which were approximately the midpoint of the age- and sex-specific RRs from the other two papers.^22,28,29^

As individuals with suspected masked hypertension have controlled office BPs (SBP/DBP 120-129/<80 mmHg), our approach likely results in more conservative RRs with antihypertensive treatment than other published literature. However, the potential reduction in CVD risk with antihypertensive treatment in masked hypertension is unknown. The reduction in high out-of-office BP with antihypertensive treatment may provide more benefit than is indicated using office BP.

**Alternate Approach to Quality-of-Life Decrements**

In the base-case analysis, the quality-of-life decrement (i.e., utility) due to CVD was additive, as in prior analyses.^14,15^ That is, the age-specific utility without CVD was reduced by a fixed amount after a CVD event occurred. For example, at age 60 years, the utility without CVD was 0.82. If a stroke occurred, the utility was reduced by 0.12 thereafter (0.82 - 0.12 = 0.70). Other published studies used a multiplicative approach (CHD = 0.79, stroke = 0.63), which generally results in larger utility reduction with CVD events.^22,28,29^ In the same example of stroke in a 60-year-old, the multiplicative approach results in a utility of 0.52 (0.82 x 0.63 = 0.52), which means that preventing a stroke increases quality-adjusted life year (QALY) gains. We therefore performed a scenario analysis using a multiplicative approach to calculating chronic utility using the same multipliers. Additionally, we varied the utility decrement associated with antihypertensive treatment (i.e., pill-taking disutility) to match the approach in other studies.^14,15,22,28^

**SUPPLEMENTAL TABLES**

**Table S1. Hypertension Treatment-Related CVD Policy Model Inputs.^14^**

| **Model Input** | **Mean** | **SD** | **Min** | **Max** | **Distribution Type** |
| --- | --- | --- | --- | --- | --- |
| Antihypertensive Medication Adherence | | | | | |
| *Probability of discontinuation within one year of initiation* | 0.430 | 0.049 | 0.340 | 0.535 | Beta |
| *Pill-taking Adherence (per number of medication classes)* | | | | | |
| 1 | 0.900 | 0.070 | 0.681 | 0.963 | Beta |
| 2 | 0.845 | 0.057 | 0.700 | 0.927 | Beta |
| 3 | 0.823 | 0.055 | 0.712 | 0.932 | Beta |
| 4+ | 0.747 | 0.053 | 0.669 | 0.881 | Beta |
| BP Changes with Treatment | | | | | |
| *BP reduction with antihypertensive treatment per full-standard dose added* | | | | | |
| Mean DBP reduction at 90 mmHg | 4.700 | 0.421 | 2.350 | 7.050 | Gamma |
| Coefficient of reduction per mmHg decrease in pretreatment DBP | 0.110 | 0.028 | 0.055 | 0.165 | Gamma |
| Mean SBP reduction at 150 mmHg | 8.700 | 0.357 | 4.350 | 13.050 | Gamma |
| Coefficient of reduction per mmHg decrease in pretreatment SBP | 0.100 | 0.025 | 0.050 | 0.150 | Gamma |
| *BP reduction with antihypertensive treatment per half-standard dose added* | | | | | |
| Mean DBP reduction at 90 mmHg | 3.700 | 0.306 | 3.100 | 4.300 | Gamma |
| Coefficient of reduction per mmHg decrease in pretreatment DBP | 0.088 | 0.022 | 0.045 | 0.132 | Gamma |
| Mean SBP reduction at 150 mmHg | 6.700 | 0.281 | 6.100 | 7.200 | Gamma |
| Coefficient of reduction per mmHg decrease in pretreatment SBP | 0.078 | 0.020 | 0.039 | 0.117 | Gamma |
| *Percent of possible BP reduction achieved with proportion of antihypertensive doses taken as directed* | | | | | |
| Polynomial regression coefficients |  |  |  |  |  |
| Intercept | 0.001 | - | - | - | - |
| 1^st^-degree | -0.135 | - | - | - | - |
| 2^nd^-degree | -0.479 | - | - | - | - |
| 3^rd^-degree | 24.244 | - | - | - | - |
| 4^th^-degree | 60.897 | - | - | - | - |
| 5^th^-degree | 59.584 | - | - | - | - |
| 6^th^-degree | 23.813 | - | - | - | - |
| 7^th^-degree | 2.494 | - | - | - | - |
| BP Measurement Accuracy | | | | | |
| *Difference between measured and “true” BP by total number of visits and measurements per visit* | | | | | |
| SBP |  |  |  |  |  |
| 1 visit with 1 measurement | 0.000 | 8.100 | - | - | Normal |
| 1 visit with ≥3 measurements | 0.000 | 6.200 | - | - | Normal |
| 2 visits with 1 measurement | 0.000 | 5.940 | - | - | Normal |
| 2 visits with ≥3 measurements | 0.000 | 4.390 | - | - | Normal |
| ≥3 visits with 1 measurement | 0.000 | 5.000 | - | - | Normal |
| ≥3 visits with ≥3 measurements | 0.000 | 3.650 | - | - | Normal |
| DBP |  |  |  |  |  |
| 1 visit with 1 measurement | 0.000 | 5.450 | - | - | Normal |
| 1 visit with ≥3 measurements | 0.000 | 4.370 | - | - | Normal |
| 2 visits with 1 measurement | 0.000 | 3.900 | - | - | Normal |
| 2 visits with ≥3 measurements | 0.000 | 3.120 | - | - | Normal |
| ≥3 visits with 1 measurement | 0.000 | 3.180 | - | - | Normal |
| ≥3 visits with ≥3 measurements | 0.000 | 2.550 | - | - | Normal |
| Adverse Events | | | | | |
| *Any adverse events by number of standard antihypertensive doses* | | | | | |
| 1 half | 0.031 | 0.024 | 0.001 | 0.093 | Beta |
| 1 full | 0.055 | 0.018 | 0.024 | 0.096 | Beta |
| 1 full + 1 half | 0.066 | 0.010 | 0.055 | 0.096 | Beta |
| 2 full | 0.087 | 0.008 | 0.073 | 0.103 | Beta |
| 2 full + 1 half | 0.101 | 0.010 | 0.087 | 0.125 | Beta |
| 3 full | 0.117 | 0.008 | 0.103 | 0.134 | Beta |
| 3 full + 1 half | 0.130 | 0.009 | 0.117 | 0.154 | Beta |
| 4 full | 0.147 | 0.008 | 0.133 | 0.164 | Beta |
| 4 full + 1 half | 0.161 | 0.010 | 0.147 | 0.186 | Beta |
| 5 full | 0.177 | 0.008 | 0.162 | 0.194 | Beta |
| *Risk of intolerable adverse events by number of antihypertensive classes* | | | | | |
| 1 class | 0.005 | 0.002 | 0.001 | 0.011 | Beta |
| 2 classes | 0.009 | 0.005 | 0.003 | 0.021 | Beta |
| 3 classes | 0.016 | 0.008 | 0.005 | 0.035 | Beta |
| 4 classes | 0.023 | 0.011 | 0.007 | 0.050 | Beta |
| 5 classes | 0.030 | 0.014 | 0.009 | 0.064 | Beta |
| *Risk of serious adverse events by number of antihypertensive classes* | | | | | |
| ≤2 classes | 0.009 | 0.001 | 0.006 | 0.010 | Beta |
| >2 classes | 0.013 | 0.002 | 0.011 | 0.017 | Beta |
| *Probability serious adverse event is fatal by age* | | | | | |
| 18-44 | 0.004 | 0.001 | 0.004 | 0.007 | Beta |
| 45-64 | 0.011 | 0.003 | 0.005 | 0.017 | Beta |
| 65-84 | 0.019 | 0.007 | 0.003 | 0.031 | Beta |
| ≥85 | 0.031 | 0.010 | 0.015 | 0.053 | Beta |

BP – blood pressure, DBP – diastolic blood pressure, SBP – systolic blood pressure, SD – standard deviation

**Table S2: Logistic Risk Functions to Estimate Probability of Incident CVD Events and Non-CVD Mortality in the CVD Policy Model.^14,15^**

| **Parameter** | **Description** | **Hazard Ratio**  **(95% UI)** | **Beta Value**  **(95% UI)** |
| --- | --- | --- | --- |
| Incident CHD event | | | |
| Age | Years | 1.107 (1.090, 1.125) | 0.10156 (0.08578, 0.11734) |
| African American | Binary | 0.885 (0.826, 0.949) | -0.12189 (-0.19158, -0.05220) |
| BMI | kg/m^2^ | 1.006 (1.000, 1.012) | 0.00597 (0.00046, 0.01147) |
| Former smoker | Binary | 1.204 (1.134, 1.278) | 0.18574 (0.12603, 0.24545) |
| Current smoker | Binary | 1.683 (1.496, 1.893) | 0.52051 (0.40291, 0.63811) |
| Cigarettes per day | - | 1.006 (1.001, 1.011) | 0.00604 (0.00126, 0.01083) |
| Systolic blood pressure | mmHg | 1.013 (1.012, 1.014) | 0.01289 (0.01149, 0.01429) |
| Diabetes | Binary | 1.916 (1.789, 2.052) | 0.65028 (0.58172, 0.71884) |
| HDL-C | mg/dL | 0.985 (0.983, 0.988) | -0.01488 (-0.01727, -0.01250) |
| LDL-C | mg/dL | 1.005 (1.005, 1.006) | 0.00543 (0.00466, 0.00619) |
| eGFR | mL/min/1.73^2^ | 0.993 (0.992, 0.995) | -0.00676 (-0.00849, -0.00504) |
| Age x current smoker | - | 0.987 (0.982, 0.991) | -0.01349 (-0.01841, -0.00856) |
| Age x systolic blood pressure | - | 1.000 (1.000, 1.000) | -0.00031 (-0.00040, -0.00021) |
| Age x diabetes | - | 0.990 (0.985, 0.995) | -0.01027 (-0.01511, -0.00544) |
| Age x HDL-C | - | 1.000 (1.000, 1.000) | 0.00033 (0.00018, 0.00049) |
| Age x LDL-C | - | 1.000 (1.000, 1.000) | -0.00019 (-0.00025, -0.00014) |
| Incident Stroke Event | | | |
| Age | Years | 1.146 (1.123, 1.170) | 0.13656 (0.11627, 0.15686) |
| African American | Binary | 1.605 (1.430, 1.802) | 0.47326 (0.35738, 0.58914) |
| Current smoker | Binary | 1.868 (1.667, 2.094) | 0.62513 (0.51121, 0.73906) |
| Systolic blood pressure | mmHg | 1.020 (1.018, 1.022) | 0.01988 (0.01773, 0.02202) |
| Diabetes | Binary | 1.950 (1.751, 2.171) | 0.66772 (0.56039, 0.77505) |
| HDL-C | mg/dL | 0.995 (0.992, 0.998) | -0.00472 (-0.00779, -0.00165) |
| LDL-C | mg/dL | 1.002 (1.000, 1.003) | 0.00172 (0.00049, 0.00295) |
| eGFR | mL/min/1.73^2^ | 0.996 (0.993, 0.998) | -0.00421 (-0.00691, -0.00152) |
| Age x African American | - | 0.977 (0.969, 0.986) | -0.02280 (-0.03126, -0.01435) |
| Age x current smoker | - | 0.990 (0.982, 0.999) | -0.00955 (-0.01772, -0.00138) |
| Age x systolic blood pressure | - | 1.000 (0.999, 1.000) | -0.00042 (-0.00056, -0.00028) |
| Age x diabetes | - | 0.984 (0.977, 0.991) | -0.01607 (-0.02356, -0.00858) |
| Non-CVD Mortality | | | |
| Age | Years | 1.104 (1.097, 1.111) | 0.09916 (0.09289, 0.10543) |
| African American | Binary | 1.501 (1.404, 1.605) | 0.40643 (0.33944, 0.47342) |
| BMI | kg/m^2^ | 0.905 (0.886, 0.925) | -0.09962 (-0.12093, -0.07832) |
| BMI^2^ | - | 1.001 (1.001, 1.002) | 0.00137 (0.00106, 0.00168) |
| Former smoker | Binary | 1.296 (1.228, 1.369) | 0.25967 (0.20511, 0.31422) |
| Current Smoker | Binary | 1.985 (1.792, 2.200) | 0.68585 (0.58327, 0.78842) |
| Cigarettes per day | Among current smokers | 1.020 (1.016, 1.025) | 0.02027 (0.01601, 0.02452) |
| Systolic blood pressure | mmHg | 1.001 (1.000, 1.002) | 0.00113 (-0.00010, 0.00236) |
| Diabetes | Binary | 1.542 (1.441, 1.650) | 0.43303 (0.36525, 0.50081) |
| eGFR | mL/min/1.73^2^ | 0.993 (0.992, 0.995) | -0.00660 (-0.00815, -0.00506) |
| Age x African American | - | 0.985 (0.980, 0.989) | -0.01530 (-0.01998, -0.01061) |
| Age x BMI^2^ | - | 1.000 (1.000, 1.000) | 0.00002 (0.00001, 0.00002) |
| Age x diabetes | - | 0.989 (0.984, 0.994) | -0.01144 (-0.01647, -0.00641) |

BMI – body mass index, CU-NHLBI – Columbia University-National Heart Lung and Blood Institute, CVD – cardiovascular disease, eGFR – Estimated Glomerular Filtration Rate, HDL-C – high-density lipoprotein cholesterol, LDL-C – low-density lipoprotein cholesterol, UI – uncertainty interval

**Table S3. CVD Risk Estimates for Masked Hypertension.**

| **Study and Data Source** | **Device and BP Threshold** | **Hazard ratio (vs. normotension) (95% CI)** |
| --- | --- | --- |
| Asayama et al. IDHOCO^3^ (n = 406) | HBPM ≥135/85 mmHg | 1.93 (1.25, 2.98) |
| Asayama et al. IDACO^4^ (n = 1,105) | Daytime ABPM ≥135/85 mmHg | 1.61 (1.27, 2.05) |
| Pierdomenico et al. Meta-analysis^8^ (n = 665) | Daytime ABPM ≥135/85 mmHg | 2.09 (1.55, 2.81) |
| Stergiou et al. IDHOCO^9^ (n = 404) | HBPM ≥135/85 mmHg (Untreated) | 1.55 (1.12, 2.14) |

ABPM – ambulatory blood pressure monitoring; CVD – cardiovascular disease; HBPM – home blood pressure monitoring; IDACO – International Database of Ambulatory Blood Pressure in Relation to Cardiovascular Outcome; IDHOCO – International Database of Home Blood Pressure in Relation to Cardiovascular Outcome

**Table S4. Probabilities for Non-Incident CVD Events in the CVD Policy Model.^15^**

| **Model Input** | **Base Case Value (%)** |
| --- | --- |
| Following CHD Event (annual probability) | |
| Recurrent CHD event within 1 year of previous CHD event, (male) | |
| 20-44 years | 3.53 |
| 45-54 years | 4.74 |
| 55-64 years | 6.49 |
| 65-74 years | 7.96 |
| 75+ years | 12.80 |
| Recurrent CHD event within 1 year of previous CHD event, (female) | |
| 20-44 years | 2.26 |
| 45-54 years | 3.96 |
| 55-64 years | 4.98 |
| 65-74 years | 8.29 |
| 75+ years | 13.55 |
| Recurrent CHD event after 1 year of previous CHD event, (male) | |
| 20-44 years | 1.22 |
| 45-54 years | 1.60 |
| 55-64 years | 2.23 |
| 65-74 years | 2.79 |
| 75+ years | 4.53 |
| Recurrent CHD event after 1 year of previous CHD event, (female) | |
| 20-44 years | 0.96 |
| 45-54 years | 1.25 |
| 55-64 years | 1.63 |
| 65-74 years | 2.72 |
| 75+ years | 4.66 |
| Stroke after CHD, (male) | |
| 20-44 years | 0.55 |
| 45-54 years | 0.55 |
| 55-64 years | 0.79 |
| 65-74 years | 0.83 |
| 75+ years | 0.92 |
| Stroke after CHD, (female) | |
| 20-44 years | 0.55 |
| 45-54 years | 0.55 |
| 55-64 years | 0.77 |
| 65-74 years | 0.87 |
| 75+ years | 0.89 |
| Following stroke event (annual probability) | |
| Recurrent stroke event | 3.60 |
| CHD after stroke within 10 years | 2.50 |
| CHD after stroke after 10 years | 2.20 |
| 30-day case fatality rates | |
| Incident CHD, (male) | |
| 20-44 years | 6.62 |
| 45-54 years | 10.31 |
| 55-64 years | 12.31 |
| 65-74 years | 14.66 |
| 75-84 years | 13.00 |
| 85+ years | 17.5.0 |
| Incident CHD, (female) | |
| 20-44 years | 5.00 |
| 45-54 years | 6.94 |
| 55-64 years | 9.29 |
| 65-74 years | 12.69 |
| 75-84 years | 10.57 |
| 85+ years | 17.09 |
| Recurrent CHD, (male) | |
| 20-44 years | 2.24 |
| 45-54 years | 7.85 |
| 55-64 years | 9.89 |
| 65-74 years | 12.96 |
| 75-84 years | 14.60 |
| 85+ years | 24.79 |
| Recurrent CHD, (female) | |
| 20-44 years | 2.22 |
| 45-54 years | 5.44 |
| 55-64 years | 6.65 |
| 65-74 years | 11.48 |
| 75-84 years | 10.95 |
| 85+ years | 24.21 |
| Any Stroke, (male) | |
| 20-44 years | 10.34 |
| 45-54 years | 9.57 |
| 55-64 years | 10.46 |
| 65-74 years | 11.36 |
| 75-84 years | 13.69 |
| 85+ years | 30.65 |
| Any stroke, (female) | |
| 20-44 years | 10.34 |
| 45-54 years | 9.57 |
| 55-64 years | 10.46 |
| 65-74 years | 11.36 |
| 75-84 years | 13.69 |
| 85+ years | 30.65 |

CHD – coronary heart disease

**Table S5. ABPM and HBPM Device Costs.**

| **Device** | **Cost (USD)** | **Source** |
| --- | --- | --- |
| ABPM | | |
| IEM 220 24HR ABPM | 1,495 | Cardiac Direct |
| QRS Opti 24 Hour ABPM | 2,195 |
| Meditech ABPM-05 | 1,799 |
| Tiba T-512 Ambulatory | 1,650 |
| Riester Ri-Cardio 24-hour ABPM | 1,799 |
| Vectracor Opti | 2,025 |
| HBPM | | |
| Beurer BM55 | 51.00 | Amazon |
| A&D UA-767F | 45.00 |
| Omron Evolv | 63.00 |

ABPM – ambulatory blood pressure monitor , HBPM – home blood pressure monitor, USD – U.S dollars.

Notes: Data pulled on June 15^th^, 2020

**Table S6. CVD Policy Model Cost and Utility Inputs.**

| **Model Input** | **Mean** | **SD** | **Source** |
| --- | --- | --- | --- |
| **Costs (2021 USD)** | | | |
| *Antihypertensive Medication (Generic), per week* | | | |
| 1 Half | 2.12 | 0.07 | Bryant et al.^14^ |
| 1 Full | 2.43 | 0.08 |
| 1 Full + 1 Half | 4.08 | 0.11 |
| 2 Full | 4.32 | 0.11 |
| 2 Full + 1 Half | 6.38 | 0.18 |
| 3 Full | 6.68 | 0.19 |
| 3 Full + 1 Half | 9.52 | 0.49 |
| 4 Full | 9.93 | 0.53 |
| 4 Full + 1 Half | 11.91 | 0.69 |
| 5 Full | 12.20 | 0.71 |
| *Background (Male), annual* | | | |
| 20-39 years | 1,747 |  | Kohli-Lynch et al.^15^ |
| 40-49 years | 3,493 |  |
| 50-59 years | 4,591 |  |
| 60-69 years | 6,118 |  |
| 70-79 years | 9,098 |  |
| 80-89 years | 13,768 |  |
| 90+ years | 26,393 |  |
| *Background (Female), annual* | | | |
| 20-39 years | 2,454 |  |  |
| 40-49 years | 4,908 |  | Kohli-Lynch et al.^15^ |
| 50-59 years | 6,660 |  |
| 60-69 years | 9,582 |  |
| 70-79 years | 11,766 |  |
| 80-89 years | 17,544 |  |
| 90+ years | 30,787 |  |
| *Acute 30-Day CHD (Male)* | | | |
| 20-39 years | 3,938 |  | Kohli-Lynch et al.^15^ |
| 40-49 years | 7,876 |  |
| 50-59 years | 13,384 |  |
| 60-69 years | 19,367 |  |
| 70-79 years | 22,848 |  |
| 80-89 years | 23,836 |  |
| 90+ years | 24,863 |  |
| *Acute 30-Day CHD (Female)* | | | |
| 20-39 years | 3,129 |  | Kohli-Lynch et al.^15^ |
| 40-49 years | 6,257 |  |
| 50-59 years | 8,402 |  |
| 60-69 years | 16,392 |  |
| 70-79 years | 20,936 |  |
| 80-89 years | 24,578 |  |
| 90+ years | 32,669 |  |
| *Acute 30-Day Stroke (Male)* | | | |
| 20-49 years | 24,780 |  | Kohli-Lynch et al.^15^ |
| 50-59 years | 21,528 |  |
| 60-69 years | 20,100 |  |
| 70-79 years | 16,963 |  |
| 80+ years | 18,126 |  |
| *Acute 30-Stroke (Female)* | | | |
| 20-49 years | 23,935 |  | Kohli-Lynch et al.^15^ |
| 50-59 years | 20,682 |  |
| 60-69 years | 19,255 |  |
| 70-79 years | 16,118 |  |
| 80+ years | 17,281 |  |
| *CHD First Year* | | | |
| 20-69 years | 12,568 |  | Kohli-Lynch et al.^15^ |
| 70+ years | 19,206 |  |
| *CHD After First Year* | | | |
| 20-89 years | 2,567 |  | Kohli-Lynch et al.^15^ |
| 90+ years | 4,036 |  |
| *Stroke First Year* | | | |
| 20+ years | 19,447 |  | Kohli-Lynch et al.^15^ |
| *Stroke After First Year* | | | |
| 20+ years | 5,404 |  | Kohli-Lynch et al.^15^ |
| *CHD Death (Male)* | | | |
| 20-21 years | 17,353 |  | Kohli-Lynch et al.^15^ |
| 22-29 years | 34,706 |  |
| 30-49 years | 60,797 |  |
| 50-59 years | 63,933 |  |
| 60-69 years | 69,512 |  |
| 70-79 years | 61,086 |  |
| 80-89 years | 51,579 |  |
| 90+ years | 44,006 |  |
| *CHD Death (Female)* | | | |
| 20-21 years | 15,491 |  | Kohli-Lynch et al.^2^ |
| 22-29 years | 30,982 |  |
| 30-39 years | 39,599 |  |
| 40-49 years | 61,181 |  |
| 50-59 years | 53,932 |  |
| 60-69 years | 65,500 |  |
| 70-79 years | 60,542 |  |
| 80-89 years | 51,737 |  |
| 90+ years | 43,815 |  |
| *Stroke Death (Male)* | | | |
| 20-49 years | 30,625 |  | Kohli-Lynch et al.^15^ |
| 50-59 years | 28,473 |  |
| 60-69 years | 27,198 |  |
| 70-79 years | 24,394 |  |
| 80+ years | 25,434 |  |
| *Stroke Death (Female)* | | | |
| 20-49 years | 30,625 |  | Kohli-Lynch et al.^15^ |
| 50-59 years | 27,717 |  |
| 60-69 years | 26,442 |  |
| 70-79 years | 23,639 |  |
| 80+ years | 24,678 |  |
| *Serious Adverse Events, per event* | | | |
| 20-34 years | 7,971 |  | Bryant et al.^14^ |
| 35-44 years | 8,480 |  |
| 45-54 years | 9,138 |  |
| 55-64 years | 9,894 |  |
| 65-74 years | 10,007 |  |
| 75-84 years | 9,835 |  |
| 85+ years | 9,003 |  |
| *Office Visit for Adverse Events, per visit* | | | |
| Intolerable | 115.52 | 10.80 | Bryant et al.^14^ CMS Physician Fee Schedule (CPT: 99214) |
| Serious | 154.78 | 14.73 | Bryant et al.^14^ CMS Physician Fee Schedule (CPT: 99215) |
| **Utilities** | | | |
| *Background* | | | |
| 20-29 years | 0.922 |  | Kohli-Lynch et al.^15^ |
| 30-39 years | 0.901 |  |
| 40-49 years | 0.871 |  |
| 50-59 years | 0.842 |  |
| 60-69 years | 0.823 |  |
| 70-79 years | 0.790 |  |
| 80+ years | 0.736 |  |
| *CHD (decrement)* | | | |
| 20-44 years | -0.0652 |  | Kohli-Lynch et al.^15^ |
| 45-54 years | -0.0626 |  |
| 55-64 years | -0.0624 |  |
| 65-74 years | -0.0628 |  |
| 75-84 years | -0.0636 |  |
| 85+ years | -0.0642 |  |
| *Stroke (decrement)* | | | |
| 20+ years | -0.1164 |  | Kohli-Lynch et al.^15^ |
| *Acute CHD* | | | |
| 20+ years | -0.1338 |  | Kohli-Lynch et al.^15^ |
| *Acute Stroke* | | | |
| 20+ years | -0.1338 |  | Kohli-Lynch et al.^15^ |

CHD – coronary heart disease, CMS – Center for Medicare and Medicaid Services, CVD – cardiovascular disease, SD – standard deviation

**Table S7. Checklist for Cost-Effectiveness Analyses from the Second Panel on Cost-Effectiveness in Health and Medicine.^36^**

| **Element** | **Manuscript** | **Technical Supplement** |
| --- | --- | --- |
| **Introduction** |  |  |
| Background of the problem | x |  |
| **Study Design and Scope** |  |  |
| Objectives | x |  |
| Audience | x |  |
| Type of analysis | x |  |
| Target populations | x |  |
| Description of interventions and comparators (including no intervention, if applicable) | x |  |
| Other intervention descriptors (e.g., care setting, model of delivery, intensity and timing of intervention) | x |  |
| Boundaries of the analysis; defining the scope or comprehensiveness of the study (e.g., for a screening program, whether only a subset of many possible strategies are included; for a transmissible condition, the extent to which disease transmission is captured; for interventions with many possible delivery settings, whether only one or more settings are modeled) | x |  |
| Time horizon | x |  |
| Analytic perspectives (e.g., reference case perspectives [health care sector, societal]; other perspectives such as employer or payer) | x |  |
| Whether this analysis meets the requirements of the reference case |  |  |
| Analysis plan | x |  |
| **Methods and Data** |  |  |
| *Trial-based analysis or model-based analysis. If model-based:* |  |  |
| Description of event pathway or model (describe condition or disease and the health states included) | x |  |
| Diagram of event pathway or model (depicting the sequencing and possible transitions among the health states included) |  | x |
| Description of model used (e.g., decision tree, state transition, microsimulation) | x |  |
| Modeling assumptions | x |  |
| Software used | x |  |
| Identification of key outcomes | x |  |
| Complete information on sources of effectiveness data, cost data, and preference weights | x | x |
| Methods for obtaining estimates of effectiveness (including approaches used for evidence synthesis) |  | x |
| Methods for obtaining estimates of costs and preference weights |  | x |
| Critique of data quality | x |  |
| Statement of costing year (i.e., the year to which all costs have been adjusted for the analysis, e.g., 2016) | x |  |
| Statement of method used to adjust costs for inflation |  |  |
| Statement of type of currency | x | x |
| Source and methods for obtaining expert judgment if applicable | N/A | N/A |
| Statement of discount rates | x |  |
| **Impact Inventory** |  |  |
| Full accounting of consequences within and outside the health care sector |  | x |
| **Results** |  |  |
| Results of model validation |  | x |
| Reference case results (discounted and undiscounted): total costs and effectiveness, incremental costs and effectiveness, incremental cost-effectiveness ratios, measures of uncertainty | x |  |
| Disaggregated results for important categories of costs, outcomes, or both | x |  |
| Results of sensitivity analysis | x | x |
| Other estimates of uncertainty | x |  |
| Graphical representation of cost-effectiveness results | x | x |
| Graphical representation of uncertainty analyses |  | x |
| Aggregate cost and effectiveness information | x |  |
| Secondary analyses | x |  |
| **Disclosures** |  |  |
| Statement of any potential conflicts of interest due to funding source, collaborations, or outside interests | x |  |
| **Discussion** |  |  |
| Summary of reference case results | x |  |
| Summary of sensitivity of results to assumptions and uncertainties in the analysis | x |  |
| Discussion of the study results in the context of results of related cost-effective analyses | x |  |
| Discussion of ethical implications (e.g., distributive implications relating to age, disability, or other characteristics of the population) |  |  |
| Limitations of the study | x |  |
| Relevance of study results to specific policy questions or decisions | x |  |

Notes: The table shows what components of the cost-effectiveness analysis checklist from the Second Panel on Cost-Effectiveness in Health and Medicine can be found in our analysis

**Table S8. Formal Health Care Sector Impact Inventory Assessment.^36^**

| **Type of impact** | **Accounted for in analysis** |
| --- | --- |
| **Health outcomes (effects)** |  |
| Longevity | x |
| Health-related-quality-of-life effects | x |
| Other health effects (e.g., adverse events) | x |
| **Medical costs** |  |
| Paid for third-party payers | x |
| Paid for by patients out-out-of-pocket | x |
| Future related medical costs (payers and patients) | x |
| Future unrelated medical costs (payers and patients) | x |

Notes: The table shows what components of the impact inventory assessment from the Second Panel on Cost Effectiveness Analyses in Health and Medicine are accounted for in our analysis.

**Table S9. Disaggregated Incremental Costs vs. Usual Care.**

| **Cost Type** | **Usual care plus HBPM** | **Usual care plus ABPM** |
| --- | --- | --- |
| Background Healthcare (95% UI) | $117 (-$86, $332) | $340 ($196, $503) |
| CVD (95% UI) | -$259 (-$344, -$186) | -$81 (-$161, $20) |
| Antihypertensive Medications (95% UI) | $653 ($563, $732) | $324 ($285, $360) |
| Physician Visits (95% UI) | -$47 (-$64, -$29) | $214 ($205, $224) |
| Treatment-Related Adverse Events (95% UI) | $582 ($510, $647) | $279 ($250, $309) |

ABPM – ambulatory blood pressure monitoring, CVD – cardiovascular disease, HBPM – home blood pressure monitoring, UI – uncertainty interval

**Table S10. Cost, Effectiveness, and Cost-effectiveness Outcomes of Modified Approach to Utility and CVD Risk Reduction with Treatment.^22,28,29^**

| **Scenario Analysis** | **Usual care plus HBPM** | **Usual care plus ABPM** |
| --- | --- | --- |
| **No pill taking disutility^28^** | | |
| Incremental Costs (2021 USD)* | $805 | $939 |
| Incremental QALYs* | 0.0341 | 0.0362 |
| ICER ($/QALY gained)^†^ | $23,591 | $64,826 |
| **Pill-taking disutility = 0.002^14,15^** | | |
| Incremental Costs (2021 USD)* | $805 | $939 |
| Incremental QALYs* | 0.0235 | 0.0310 |
| ICER ($/QALY gained)^†^ | Extendedly dominated | $30,269 |
| **Pill-taking disutility = 0.006^22^** | | |
| Incremental Costs (2021 USD)* | $805 | $939 |
| Incremental QALYs* | 0.0024 | 0.0207 |
| ICER ($/QALY gained)^†^ | Extendedly dominated | $45,377 |

ABPM – ambulatory blood pressure monitor, CVD – cardiovascular disease, HBPM – home blood pressure monitor, ICER – incremental cost-effetiveness ratio, QALY – quality-adjusted life year, USD – U.S. dollars.

*Relative to usual care

^†^Relative to next least costly, non-dominated strategy

Notes: In this scenario analysis, we modified the relative risk (RR) of a CVD event with antihypertensive treatment and the chronic utility decrements after CVD events to be comparable to the approach used in other studies examining the cost-effectiveness of ABPM and HBPM (**Supplemental Methods**).^22,28,29^ In the no pill taking disutility scenario, the ICER for Usual care plus ABPM vs. Usual care alone was $25,939 per QALY gained.

**Table S11. Cost, Effectiveness, and Cost-effectiveness Outcomes of Scenario Analyses.**

| **Scenario Analysis** | **Usual care plus HBPM** | **Usual care plus ABPM** |
| --- | --- | --- |
| **HBPM device supplied by clinic (HBPM used by 52 patients/year)** | | |
| Incremental Costs (2021 USD)* | $992 | $1,076 |
| Incremental QALYs* | -0.0021 | 0.0126 |
| ICER ($/QALY gained)^†^ | Dominated by usual care | $85,165 |
| **HBPM sensitivity and specificity = 100%** | | |
| Incremental QALYs* | $1,002 | $1,076 |
| ICER ($/QALY gained)^†^ | 0.0126 | 0.0126 |
| Incremental QALYs* | $79,353 | Dominated by usual care plus HBPM |
| **ABPM Sensitivity = 91.8%, Specificity = 41.4% (i.e., same diagnostic accuracy as HBPM)** | | |
| Incremental QALYs* | $1,046 | $1,057 |
| ICER ($/QALY gained)^†^ | -0.0022 | -0.0042 |
| Incremental QALYs* | Dominated by usual care | Dominated by usual care |

ABPM – ambulatory blood pressure monitor, BP – blood pressure, CVD – cardiovascular disease, HBPM – home blood pressure monitor, ICER – incremental cost-effetiveness ratio, QALY – quality-adjusted life year, USD – U.S. dollars.

*Relative to usual care

^†^Relative to next least costly, non-dominated strategy

**SUPPLEMENTAL FIGURES**

**Figure S1. Diagram of CVD Policy Model.**

Non-CVD or CVD-related Death

CHD

No CVD

CHD + Stroke

Stroke

CHD – coronary heart disease, CVD – cardiovascular disease

Notes: The figure shows the structure of the CVD Policy Model. Each arrow represents a health state transition. Individuals can only change health states once per cycle (year).

**Figure S2. Model Validation and Calibration Results.**

| **(a) CHD Incidence** | **(b) Total CHD Event Rate** |
| --- | --- |
| **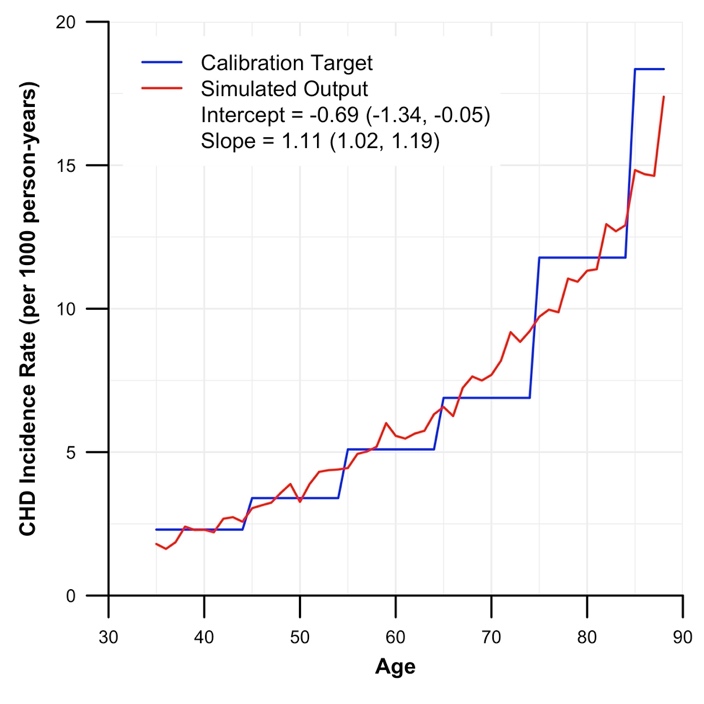** | **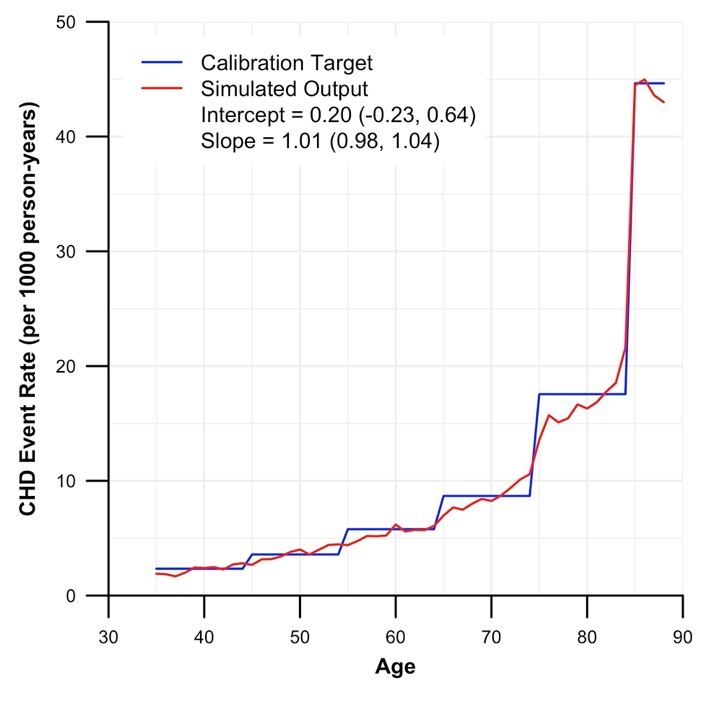** |
| **(c) CHD Mortality Rate** | **(d) Stroke Incidence** |
| **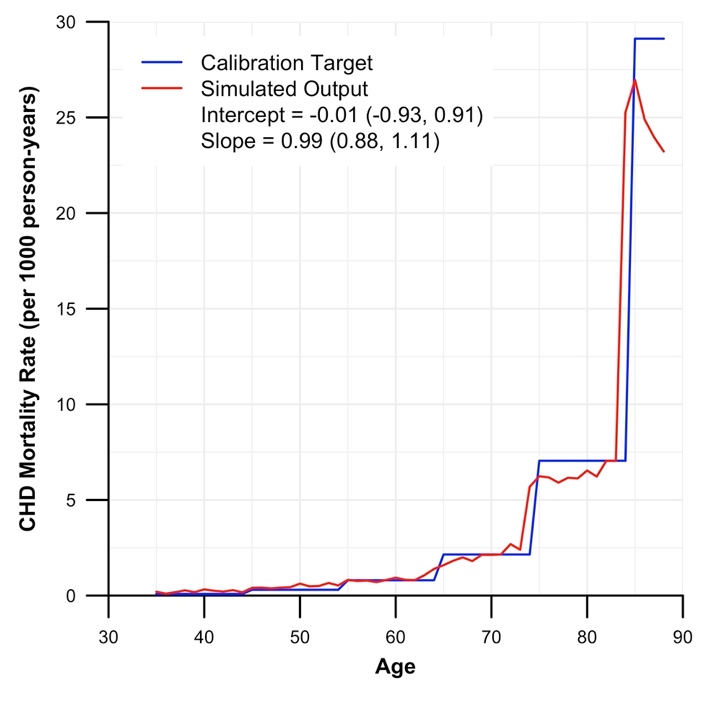** | **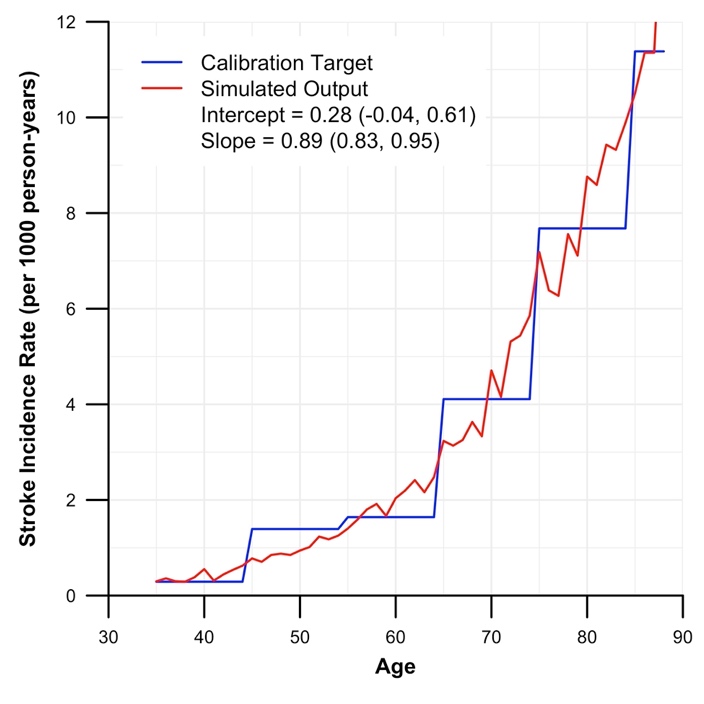** |

| **(e) Total Stroke Event Rate** | **(f) Stroke Mortality Rate** |
| --- | --- |
| **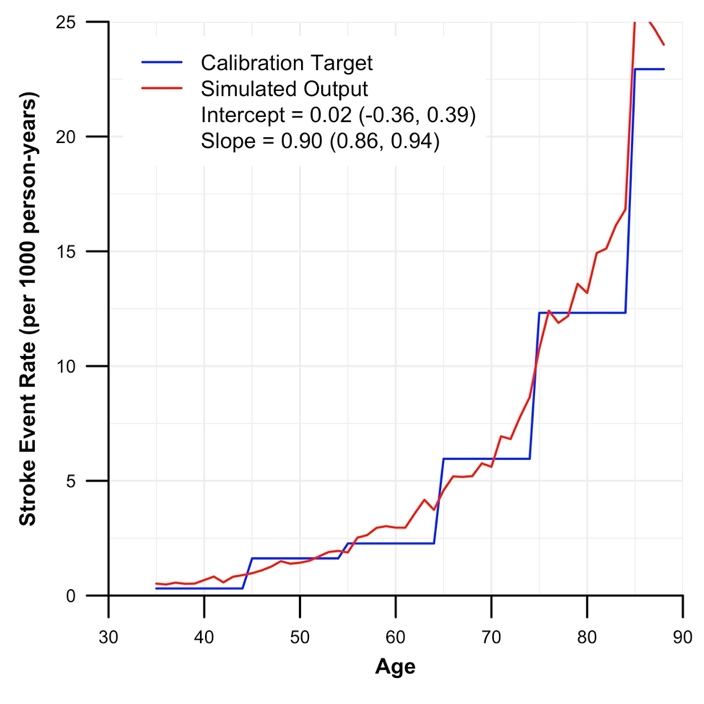** | **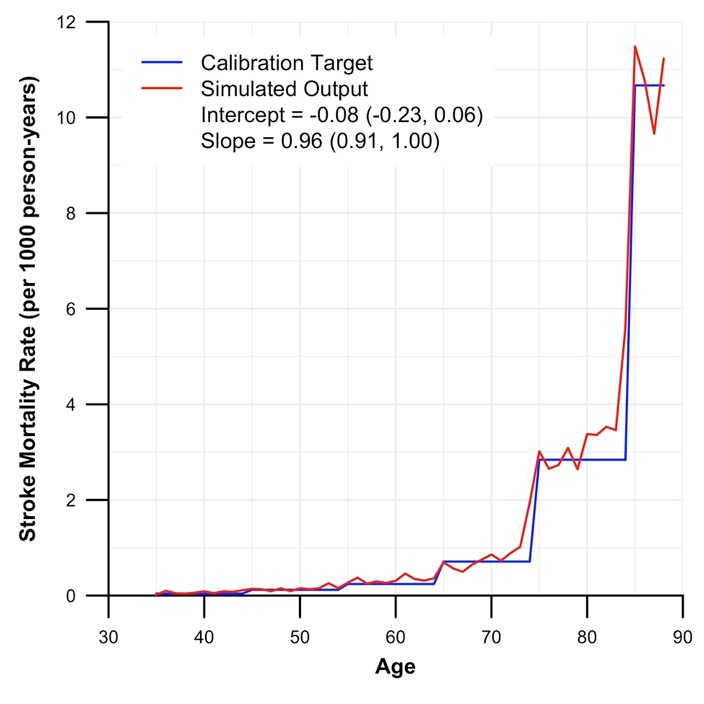** |
| **(g) All-Cause Mortality Rate** |  |
| **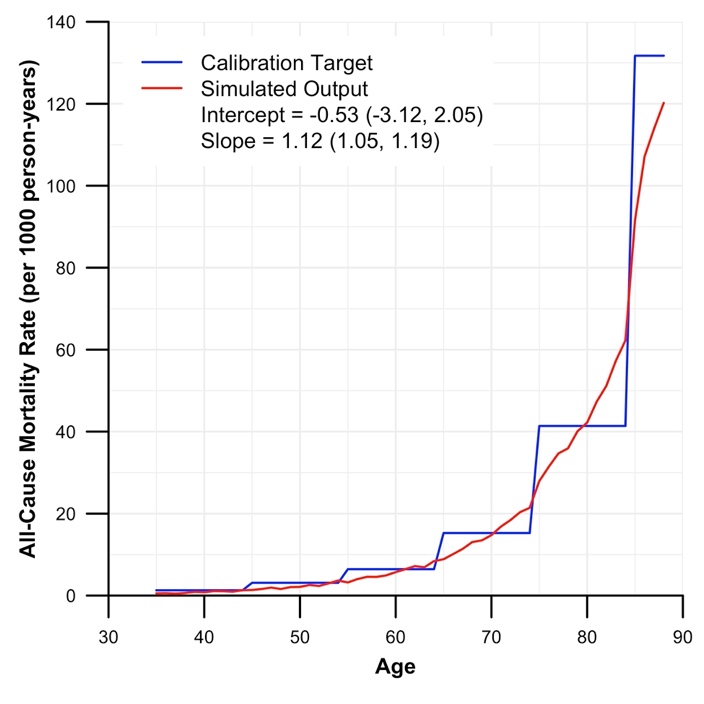** |  |

CHD – coronary heart disease, CVD – cardiovascular disease

Notes: Calibration targets represent contemporary incidence and total event rates of CHD and stroke, and CVD and all-cause mortality from the Centers for Disease Control and Prevention, National Hospital Discharge Survey, National Inpatient Sample, and National Vital Statistics System. The intercept and slope were derived for the target vs. simulated values with the 95% confidence interval shown in parentheses.

**Figure S3. Cumulative Incidence of CVD Events.**

| **(a) CHD** | **(b) Stroke** |
| --- | --- |
| **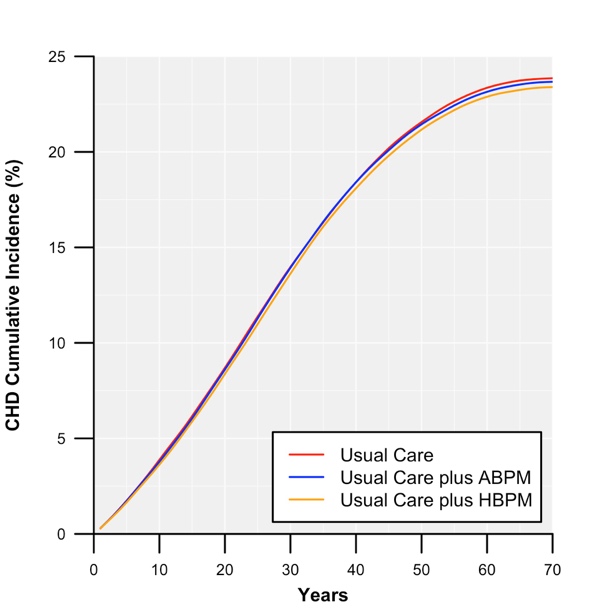** | 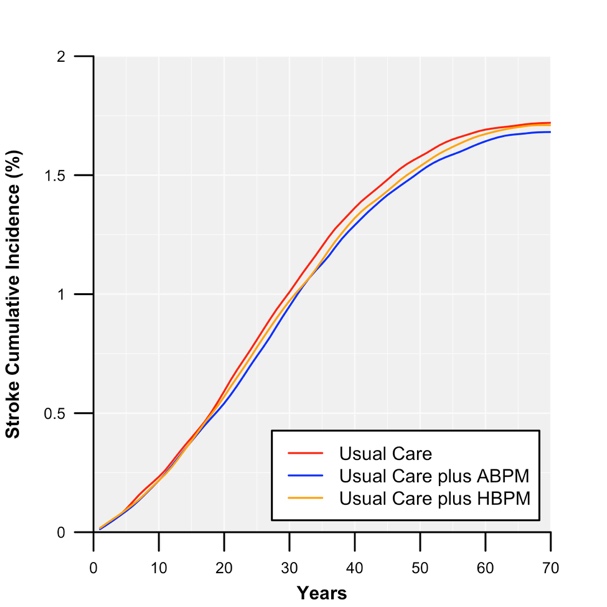 |

ABPM – ambulatory blood pressure monitoring, CHD – Coronary heart disease, HBPM – home blood pressure monitoring

**Figure S4. Cost-Effectiveness Scatter Plot (vs. Usual Care alone).**

**
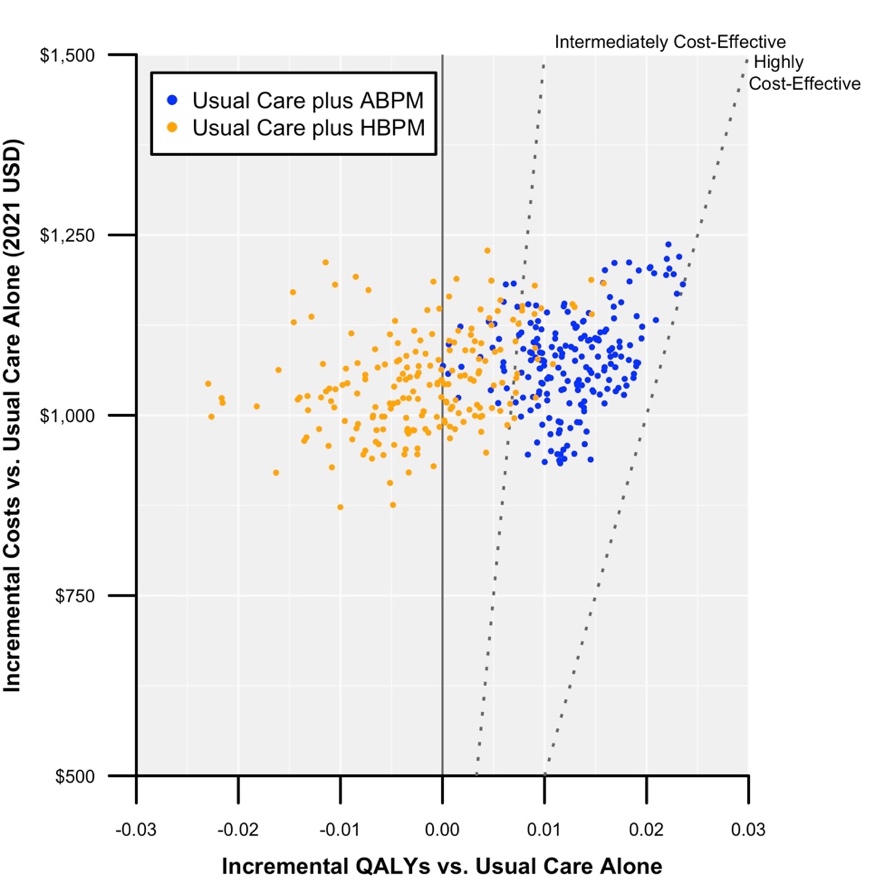
**

ABPM – ambulatory blood pressure monitoring, HBPM – home blood pressure monitoring, QALY – quality adjusted life year, USD – U.S dollar

Notes: The figure shows the results of 100 probabilistic simulations, where the model parameters were simultaneously varied by randomly sampling values from pre-specified statistical distributions. The x-axis shows the incremental QALYs relative to usual care, and the y-axis the incremental total direct healthcare costs relative to usual care. The dotted lines represent the cost-effectiveness thresholds vs. usual care of <$50,000/QALY gained (i.e., highly cost-effective) and <$150,000/QALY gained (i.e., at least intermediately cost-effective).

**Figure S5. Two-Way Sensitivity Analysis of Reducing the Risk of Serious Adverse Events and Relative Risk for CVD Events with Antihypertensive Treatment.**

| **ICER ($/QALY gained)** | **Assessment** |
| --- | --- |
| ≥$150,000 | Not Cost-Effective |
| $50,000 to <$150,000 | Intermediately Cost-Effective |
| <$50,000 | Highly Cost-Effective |

**A) Usual Care plus ABPM vs. Usual Care Alone**

| **Percent Reduction in RR for CVD** | **Percent reduction in probability of treatment-related SAEs** | | | | | |
| --- | --- | --- | --- | --- | --- | --- |
|  | 0%  (base case) | 10% | 20% | 30% | 40% | 50% |
| 0%  (base case) | $85,164 | $96,138 | $118,095 | $105,723 | $105,712 | $103,670 |
| 10% | $65,394 | $68,821 | $75,485 | $69,140 | $68,250 | $69,765 |
| 20% | $48,151 | $48,903 | $50,193 | $47,145 | $45,058 | $45,402 |
| 30% | $38,629 | $38,380 | $37,903 | $35,756 | $34,631 | $34,839 |
| 40% | $31,186 | $31,058 | $30,205 | $28,818 | $27,785 | $28,085 |
| 50% | $25,668 | $24,889 | $23,674 | $22,520 | $21,101 | $21,202 |

**B) Usual Care plus ABPM vs. Usual Care plus HBPM**

| **Percent Reduction in RR for CVD** | **Percent reduction in probability of treatment-related SAEs** | | | | | |
| --- | --- | --- | --- | --- | --- | --- |
|  | 0%  (base case) | 10% | 20% | 30% | 40% | 50% |
| 0%  (base case) | $2,046* | $2,430* | $1,826* | $10,206* | $11,211* | $14,586* |
| 10% | $6,305^†^ | $7,035^†^ | $8,177^†^ | $14,503* | $14,929* | $18,284* |
| 20% | $9,769^†^ | $11,944^†^ | $14,413^†^ | $18,435^†^ | $18,115^†^ | $21,302^†^ |
| 30% | $13,319^†^ | $16,584^†^ | $20,962^†^ | $22,681^†^ | $22,611^†^ | $25,601^†^ |
| 40% | $18,331^†^ | $23,184^†^ | $30,720 | $28,478^†^ | $28,033 | $30,696 |
| 50% | $21,967^†^ | $27,657 | $42,633 | $35,074 | $34,495 | $36,570 |

ABPM – Ambulatory blood pressure monitoring, CVD – cardiovascular disease, HBPM – home blood pressure monitoring, ICER – incremental cost-effectiveness ratio, RR – relative risk, SAE – serious adverse events.

*Usual care plus HBPM is dominated by usual care (i.e., usual care was less costly and more effective than usual care plus HBPM).

^†^When including all three strategies (i.e., usual care, usual care plus ABPM, and usual care plus HBPM), usual care plus HBPM was extendedly dominated by usual care plus ABPM (i.e., usual care plus ABPM gained more QALYs at a lower cost per QALY gained than usual care plus HBPM).

Notes: The figure shows the ICER for Usual Care plus ABPM vs. Usual Care (**Panel A**) and Usual Care plus ABPM vs. Usual Care plus HBPM (**Panel B**) when simultaneously varying the RR for CVD events with antihypertensive treatment and the probability of treatment-related SAEs, while holding all other model parameters constant. The RR for CVD events was per 10-mmHg reduction in SBP; base case RR was 0.82 for coronary heart disease events and 0.70 for stroke. The base case probability of treatment-related SAEs requiring hospitalization was 0.9% when using ≤2 antihypertensive medication classes and 1.3% when using >2 classes. The CVD RRs and probability of SAEs were reduced by the percentages indicated (e.g., 50% reduction in the RR of CHD with treatment, 0.82 x 50% = 0.41 per 10 mmHg reduction in SBP).

**Figure S6. Cumulative Incidence of CVD events Using Modified Approach to CVD Risk Reduction with Antihypertensive Treatment.**

| **(a) CHD** | **(b) Stroke** |
| --- | --- |
| **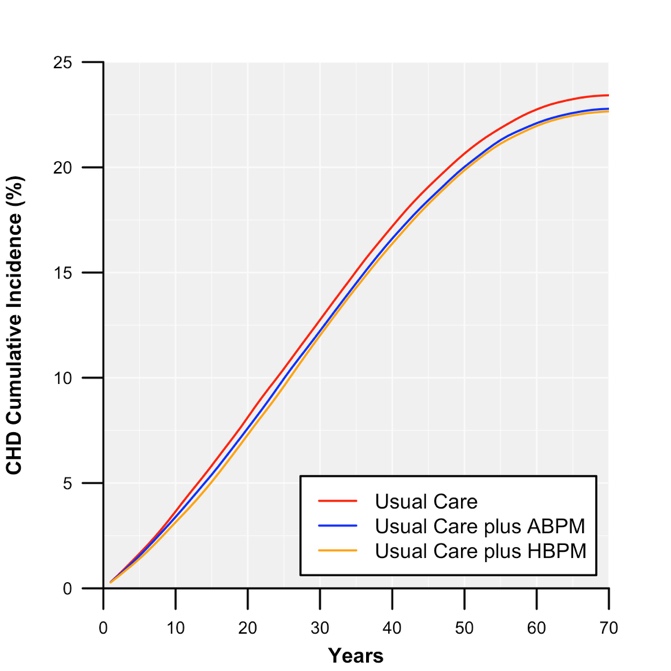** | **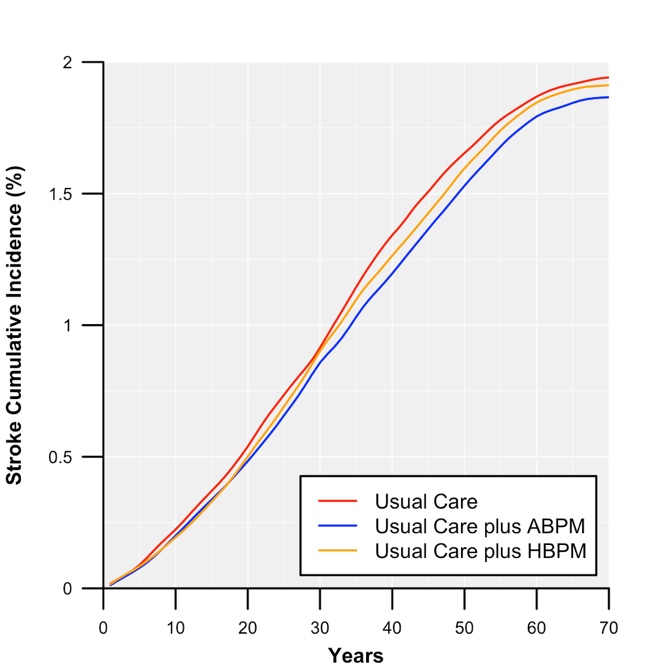** |

ABPM – ambulatory blood pressure monitoring, CHD – coronary heart disease, CVD – cardiovascular disease, HBPM – home blood pressure monitoring

Notes: In this scenario analysis, the relative risk (RR) of a CVD event with antihypertensive treatment was modified to be comparable to the approach used in other studies examining the cost-effectiveness of ABPM and HBPM.^22,28,29^

**REFERENCES**

1. Whelton PK, Carey RM, Aronow WS, et al. 2017 ACC/AHA/AAPA/ABC/ACPM/AGS/APhA/ASH/ASPC/NMA/PCNA Guideline for the Prevention, Detection, Evaluation, and Management of High Blood Pressure in Adults: Executive Summary: A Report of the American College of Cardiology/American Heart Association Task Force on Clinical Practice Guidelines. *Circulation.* 2018;138(17):e426-e483.

2. Asayama K, Thijs L, Brguljan-Hitij J, et al. Risk stratification by self-measured home blood pressure across categories of conventional blood pressure: a participant-level meta-analysis. *PLoS Med.* 2014;11(1):e1001591.

3. Asayama K, Thijs L, Li Y, et al. Setting thresholds to varying blood pressure monitoring intervals differentially affects risk estimates associated with white-coat and masked hypertension in the population. *Hypertension.* 2014;64(5):935-942.

4. Pierdomenico SD, Cuccurullo F. Prognostic value of white-coat and masked hypertension diagnosed by ambulatory monitoring in initially untreated subjects: an updated meta analysis. *Am J Hypertens.* 2011;24(1):52-58.

5. Stergiou GS, Asayama K, Thijs L, et al. Prognosis of white-coat and masked hypertension: International Database of HOme blood pressure in relation to Cardiovascular Outcome. *Hypertension.* 2014;63(4):675-682.

6. Wang YC, Shimbo D, Muntner P, Moran AE, Krakoff LR, Schwartz JE. Prevalence of Masked Hypertension Among US Adults With Nonelevated Clinic Blood Pressure. *Am J Epidemiol.* 2017;185(3):194-202.

7. Williams B, Mancia G, Spiering W, et al. 2018 ESC/ESH Guidelines for the management of arterial hypertension: The Task Force for the management of arterial hypertension of the European Society of Cardiology and the European Society of Hypertension: The Task Force for the management of arterial hypertension of the European Society of Cardiology and the European Society of Hypertension. *J Hypertens.* 2018;36(10):1953-2041.

8. Anstey DE, Pugliese D, Abdalla M, Bello NA, Givens R, Shimbo D. An Update on Masked Hypertension. *Curr Hypertens Rep.* 2017;19(12):94.

9. Ettehad D, Emdin CA, Kiran A, et al. Blood pressure lowering for prevention of cardiovascular disease and death: a systematic review and meta-analysis. *Lancet.* 2016;387(10022):957-967.

10. Muntner P, Shimbo D, Carey RM, et al. Measurement of Blood Pressure in Humans: A Scientific Statement From the American Heart Association. *Hypertension.* 2019;73(5):e35-e66.

11. Anstey DE, Bradley C, Shimbo D. USPSTF Recommendation Statement on Hypertension Screening in Adults-Where Do We Go From Here? *JAMA Netw Open.* 2021;4(4):e214203.

12. US Preventive Services Task Force, Krist AH, Davidson KW, et al. Screening for Hypertension in Adults: US Preventive Services Task Force Reaffirmation Recommendation Statement. *JAMA.* 2021;325(16):1650-1656.

13. Siu AL, Force USPST. Screening for high blood pressure in adults: U.S. Preventive Services Task Force recommendation statement. *Ann Intern Med.* 2015;163(10):778-786.

14. Bryant KB, Moran AE, Kazi DS, et al. Cost-Effectiveness of Hypertension Treatment by Pharmacists in Black Barbershops. *Circulation.* 2021;143(24):2384-2394.

15. Kohli-Lynch CN, Bellows BK, Thanassoulis G, et al. Cost-effectiveness of Low-density Lipoprotein Cholesterol Level-Guided Statin Treatment in Patients With Borderline Cardiovascular Risk. *JAMA Cardiol.* 2019;4(10):969-977.

16. Moran AE, Odden MC, Thanataveerat A, et al. Cost-effectiveness of hypertension therapy according to 2014 guidelines. *N Engl J Med.* 2015;372(5):447-455.

17. Weinstein MC, Coxson PG, Williams LW, Pass TM, Stason WB, Goldman L. Forecasting coronary heart disease incidence, mortality, and cost: the Coronary Heart Disease Policy Model. *Am J Public Health.* 1987;77(11):1417-1426.

18. Cohen LP, Vittinghoff E, Pletcher MJ, et al. Association of Midlife Cardiovascular Risk Factors With the Risk of Heart Failure Subtypes Later in Life. *J Card Fail.* 2021;27(4):435-444.

19. Oelsner EC, Balte PP, Cassano PA, et al. Harmonization of Respiratory Data From 9 US Population-Based Cohorts: The NHLBI Pooled Cohorts Study. *Am J Epidemiol.* 2018;187(11):2265-2278.

20. Zeki Al Hazzouri A, Vittinghoff E, Zhang Y, et al. Use of a pooled cohort to impute cardiovascular disease risk factors across the adult life course. *Int J Epidemiol.* 2019;48(3):1004-1013.

21. Zhang Y, Vittinghoff E, Pletcher MJ, et al. Associations of Blood Pressure and Cholesterol Levels During Young Adulthood With Later Cardiovascular Events. *J Am Coll Cardiol.* 2019;74(3):330-341.

22. Beyhaghi H, Viera AJ. Comparative Cost-Effectiveness of Clinic, Home, or Ambulatory Blood Pressure Measurement for Hypertension Diagnosis in US Adults. *Hypertension.* 2019;73(1):121-131.

23. Bryant KB, Sheppard JP, Ruiz-Negron N, et al. Impact of Self-Monitoring of Blood Pressure on Processes of Hypertension Care and Long-Term Blood Pressure Control. *J Am Heart Assoc.* 2020;9(15):e016174.

24. Kronish IM, Edmondson D, Shimbo D, Shaffer JA, Krakoff LR, Schwartz JE. A Comparison of the Diagnostic Accuracy of Common Office Blood Pressure Measurement Protocols. *Am J Hypertens.* 2018;31(7):827-834.

25. Sheppard JP, Stevens R, Gill P, et al. Predicting Out-of-Office Blood Pressure in the Clinic (PROOF-BP): Derivation and Validation of a Tool to Improve the Accuracy of Blood Pressure Measurement in Clinical Practice. *Hypertension.* 2016;67(5):941-950.

26. Sheppard JP, Martin U, Gill P, et al. Prospective external validation of the Predicting Out-of-OFfice Blood Pressure (PROOF-BP) strategy for triaging ambulatory monitoring in the diagnosis and management of hypertension: observational cohort study. *BMJ.* 2018;361:k2478.

27. Hodgkinson J, Mant J, Martin U, et al. Relative effectiveness of clinic and home blood pressure monitoring compared with ambulatory blood pressure monitoring in diagnosis of hypertension: systematic review. *BMJ.* 2011;342:d3621.

28. Lovibond K, Jowett S, Barton P, et al. Cost-effectiveness of options for the diagnosis of high blood pressure in primary care: a modelling study. *Lancet.* 2011;378(9798):1219-1230.

29. Monahan M, Jowett S, Lovibond K, et al. Predicting Out-of-Office Blood Pressure in the Clinic for the Diagnosis of Hypertension in Primary Care: An Economic Evaluation. *Hypertension.* 2018;71(2):250-261.

30. Law M, Wald N, Morris J. Lowering blood pressure to prevent myocardial infarction and stroke: a new preventive strategy. *Health Technol Assess.* 2003;7(31):1-94.

31. Xie X, Atkins E, Lv J, et al. Effects of intensive blood pressure lowering on cardiovascular and renal outcomes: updated systematic review and meta-analysis. *Lancet.* 2016;387(10017):435-443.

32. Law MR, Wald NJ, Morris JK, Jordan RE. Value of low dose combination treatment with blood pressure lowering drugs: analysis of 354 randomised trials. *BMJ.* 2003;326(7404):1427.

33. SPRINT Research Group, Wright JT, Jr., Williamson JD, et al. A Randomized Trial of Intensive versus Standard Blood-Pressure Control. *N Engl J Med.* 2015;373(22):2103-2116.

34. Hodgkinson JA, Lee MM, Milner S, et al. Accuracy of blood-pressure monitors owned by patients with hypertension (ACCU-RATE study): a cross-sectional, observational study in central England. *Br J Gen Pract.* 2020;70(697):e548-e554.

35. Gold MR, Stevenson D, Fryback DG. HALYS and QALYS and DALYS, Oh My: similarities and differences in summary measures of population Health. *Annu Rev Public Health.* 2002;23:115-134.

36. Sanders GD, Neumann PJ, Basu A, et al. Recommendations for Conduct, Methodological Practices, and Reporting of Cost-effectiveness Analyses: Second Panel on Cost-Effectiveness in Health and Medicine. *JAMA.* 2016;316(10):1093-1103.

37. Anderson JL, Heidenreich PA, Barnett PG, et al. ACC/AHA statement on cost/value methodology in clinical practice guidelines and performance measures: a report of the American College of Cardiology/American Heart Association Task Force on Performance Measures and Task Force on Practice Guidelines. *J Am Coll Cardiol.* 2014;63(21):2304-2322.

38. Urbich M, Globe G, Pantiri K, et al. A Systematic Review of Medical Costs Associated with Heart Failure in the USA (2014-2020). *Pharmacoeconomics.* 2020;38(11):1219-1236.

39. Virani SS, Alonso A, Aparicio HJ, et al. Heart Disease and Stroke Statistics-2021 Update: A Report From the American Heart Association. *Circulation.* 2021;143(8):e254-e743.

40. Schwartz JE, Muntner P, Kronish IM, et al. Reliability of Office, Home, and Ambulatory Blood Pressure Measurements and Correlation With Left Ventricular Mass. *J Am Coll Cardiol.* 2020;76(25):2911-2922.
